# Pharmacokinetics of Snake Antivenom Following Intravenous and Intramuscular Administration in Envenomed Large Animal Model

**DOI:** 10.3390/pharmaceutics17020212

**Published:** 2025-02-07

**Authors:** Erika Gamulin, Sanja Mateljak Lukačević, Maja Lang Balija, Ana Smajlović, Dražen Vnuk, Jadranka Gulan Harcet, Maja Tomičić, Ana Hećimović, Beata Halassy, Tihana Kurtović

**Affiliations:** 1 Centre for Research and Knowledge Transfer in Biotechnology, University of Zagreb, Rockefellerova 10, HR-10000 Zagreb, Croatia; egamulin@unizg.hr (E.G.); smatelja@unizg.hr (S.M.L.); mlbalija@unizg.hr (M.L.B.); bhalassy@unizg.hr (B.H.); 2 Clinic for Surgery, Orthopaedics and Ophthalmology, Faculty of Veterinary Medicine, University of Zagreb, Heinzelova 55, HR-10000 Zagreb, Croatia; asmajlovic@vef.unizg.hr (A.S.); dvnuk@vef.unizg.hr (D.V.); 3 Croatian Institute of Transfusion Medicine, Petrova 3, HR-10000 Zagreb, Croatia; jadranka.gulan-harcet@hztm.hr (J.G.H.); maja.tomicic@hztm.hr (M.T.); ana.hecimovic@hztm.hr (A.H.)

**Keywords:** Zagreb antivenom, *Vipera ammodytes* venom, administration route, pharmacokinetics, lymphatic system, systemic circulation, immunotherapy, drug efficacy

## Abstract

**Background**: The parenteral administration of antivenoms is the mainstay in snakebite envenoming therapy. The standardized protocol does not exist, but it is agreed that the intravenous (*i.v.*) route is more effective than the others, especially the intramuscular (*i.m.*) route, based on the monitoring of venom/antivenom pharmacokinetics in the systemic circulation. Recent evidence suggests that the lymphatic system may be crucial in abolishing venom action. **Methods:** A preclinical study was performed to determine the optimal administration route with emphasis on venom/antivenom interplay in both the blood and lymph of experimentally envenomed sheep. Timed level measurements were used to compare the antivenom effect on the decrement of venom quantities in both relevant body compartments. Hematological and coagulation parameters, as well as proportions of developed anti-antivenom IgGs, were evaluated. **Results:** The *i.m.* antivenom resulted in faster and greater lymphatic absorption and complete neutralization of the venom, whereas the *i.v.* antivenom only slowed its absorption. The total amount of venom reaching the lymph (AUC_0-*t*_) was two times lower after *i.m.* administration. In the systemic circulation, *i.m.* antivenom had a lower peak concentration (*c*_max_) and a longer time to reach it (*t*_max_). However, the total venom exposure was three times lower than with *i.v.* antivenom. Irrespective of the treatment approach, both groups showed improvement in blood disorders with no significant difference in humoral response against equine F(ab’)_2_ fragments. **Conclusions:** *I.m.* administration proved to be a viable option for the snakebite management.

## 1. Introduction

Snakebite envenoming is a major public health burden [1,2]. Approximately 2.7 million people suffer from its consequences each year, and more than 400,000 of those who survive remain permanently disabled. The World Health Organization (WHO) has developed a comprehensive strategy to reduce the devastating effects of envenoming by ensuring global access to safe and effective antivenoms as the only specific and validated life-saving therapeutics. Antivenoms are produced from animal-derived hyperimmune plasma and consist of whole IgGs or their derivative products (Fab or F(ab’)_2_ fragments), which are capable of neutralizing and reversing lethal and tissue-damaging toxic effects in envenomed individuals [3]. Their parenteral administration still remains the mainstay of envenoming therapy [4], a principle that dates back to the 19th century [5]. Today, the entire world faces a critical and long-standing shortage of antivenoms, primarily affecting low-income [6,7,8,9], but also high-income, countries [10,11,12], whose alleviation requires the development of feasible and profitable production strategies, rational use and the implementation of well-designed treatment protocols [6,13,14].

The efficacy of antivenom-based immunotherapy depends on the ability of venom-specific antibodies to find themselves in the same distribution space as the venom components, redistribute and ultimately eliminate them, thereby avoiding any adverse effects [15]. Beyond the preclinical neutralization potential [16], the efficacy of antivenoms is influenced by several factors. The route of administration is emerging as one of the most important [17]. There is no standardized protocol for antivenom administration across Europe, similarly as in many other regions of the world [18]. It is a WHO recommendation that, whenever possible, snake antivenoms should preferably be given intravenously (*i.v.*) [19] as a principle of harmonization of their pharmacokinetics with that of the target venom, since this should eliminate the restraint associated with the intramuscular (*i.m.*) route [13,20]. Until recently, almost all studies of venom and antivenom pharmacokinetics have been directed solely toward time measurements in the systemic circulation [21,22,23,24,25,26,27]. It appeared that antivenoms administered *i.m.* reach the bloodstream slowly and inefficiently, with a significantly longer time required to reach the maximum concentration, followed by poor bioavailability [21,23,28]. Such pharmacokinetic behavior is not adequately adapted, both in terms of timing and quantity, to the much faster arrival of subcutaneous (*s.c.*) or *i.m.* venom to the bloodstream [29,30]. On the contrary, with *i.v.* application, the entire antivenom fraction is immediately available in the systemic circulation, allowing for the prompt neutralization of rapidly absorbed venom components [31,32]. As a result, the widespread belief that *i.v.* application is more effective than *i.m.* has been established. Accordingly, it has been given priority by authorities despite the lack of randomized controlled trials. The WHO advises the *i.m.* route only as a substitute strategy at peripheral first-aid stations far from medical care and in the cases where *i.v.* access has proved impossible, as it can cause extreme pain and an increased risk of intracompartmental pressure [19]. Nevertheless, the *i.m.* route is still widely used as a first-aid measure in snakebite management, with a number of manufacturers prescribing their products for *i.m.* and/or *s.c.* application [33,34,35,36,37,38] and publications confirming its positive influence on the clinical outcome [39,40,41,42].

However, *i.m.* administration may not be such an incomprehensible concept if one considers that snake venoms are complex mixtures of proteins of variable molecular weight [43], which, in most envenomation cases, are injected into the interstitial space either by the *i.m.* or the *s.c.* route, and whose absorption into the bloodstream may occur via blood or lymph capillaries, depending on their size [44]. It is generally accepted that those of viperids are primarily absorbed through the lymphatic system, which serves as a depot for their continuous release into the systemic circulation [45]. This lymphatic absorption acts as a ”gateway“ for the entry and distribution of venom components, influencing their systemic availability and ability to reach target organs [46]. Low-molecular-weight elapid toxins are known for their ability to bypass the lymphatic system [28,47]. They can be delivered directly from the injection site into the bloodstream and reach their sites of action in a very short time. Unlike venoms, antivenoms have a uniform composition containing only large molecules. Therefore, when administered *i.m.*, they can reach the central compartment only by slow diffusion into the initial lymphatics. Here, immediate contact with the venom coming from the injection site to the lymph and timely prevention of its escape into the bloodstream can be achieved.

In fact, as demonstrated recently, *i.v.* antivenom-mediated neutralization occurs not only in the systemic circulation but also in the lymphatic system [48]. In particular, there is evidence that the lymphatic system not only plays a role in venom distribution and bioavailability but also serves as a compartment where antivenom, extravasated from the blood after *i.v.* administration, eliminates a large share of venom before the lymph reaches the systemic circulation [48]. Considering that the antivenom may have substantial neutralizing activity in the lymphatic system, the matching of venom/antivenom pharmacokinetics in the systemic circulation may not be the best, or at least only, indicator of therapeutic success. The role of *i.m.* antivenom in the elimination of lymph-absorbed venom may be even greater, but it has not been studied, highlighting an important gap in existing knowledge. More importantly, considering the relevance of both body compartments, blood and lymph, *i.v.* and *i.m.* administration routes have not been compared in a consolidated manner.

The main objective was to compare two common routes of antivenom administration in a comprehensive preclinical study involving F(ab’)_2_ antivenom specific for the venom of *Vipera ammodytes*, a species of high medical importance in Southeastern Europe [49], which causes severe envenomation, local edema and coagulation disorders [32] and determines which treatment approach is more effective in overcoming either the whole venom or its neurotoxic fraction, consisting of ammodytoxins (Atxs) in both relevant body compartments where neutralization occurs. To address the issue, sheep were employed as an experimental model suitable for studying the lymphatic absorption of *s.c.* injected proteins with results comparable to those in humans [46,50]. Their size facilitates the performance of surgical techniques and the collection of biological samples in the required quantities without compromising their health [51,52]. The second objective was to provide information on the effectiveness of *i.m.* and *i.v.* antivenoms in terms of their ability to reverse disturbances affecting the main hematological and coagulation parameters after experimentally induced envenoming. The final objective was to give an insight into the safety profiles of *i.m.* and *i.v.* antivenoms, as assessed by the evaluation of antibodies raised against equine F(ab’)_2_ fragments.

In summary, this study will either provide additional evidence for the *i.v.* route precedence or disprove the negative connotation associated with the *i.m.* administration, proving it to be an equally good or even better concept from the standpoint of preclinical assessment. Additionally, the platform here described may provide a valuable tool for the design of new or improved treatment protocols, not only for snakebite management but also for other medical conditions targeted by immunotherapeutics, particularly those based on monoclonal antibodies, whose breakthrough into clinical practice is now more evident than ever, offering the opportunity to create more effective and safer strategies for the enhancement of patient outcomes.

## 2. Materials and Methods

### 2.1. Chemical, Reagents, Snake Venom and Antivenom

The rabbit anti-horse IgG conjugated with horseradish peroxidase (HRP-anti-horse IgG) (product number: A6917-1ML; batch number: 0000161499) and the rabbit anti-guinea pig IgG conjugated with horseradish peroxidase (HRP-anti-guinea pig IgG) (product number: A5545-BULK; batch number: 0000119340) were obtained from Sigma-Aldrich (St. Louis, MO, USA). The horseradish peroxidase-conjugated goat anti-equine F(ab’)_2_ antibody (HRP-anti-equine F(ab’)_2_ IgG) (product number: ABIN101430; batch number: 45751) and rabbit anti-sheep IgG (HRP-anti-ovine IgG) (product number: ABIN102255; batch number: 34337) were from Antibodies Online (Aachen, Germany). *O*-phenylenediamine dihydrochloride (OPD), Tween 20 and bovine serum albumin (BSA) were from Sigma-Aldrich (St. Louis, MO, USA). Chemicals for buffers and solutions were from AppliChem (Darmstadt, Germany) unless stated otherwise. *V. ammodytes ammodytes* (*Vaa*) venom was collected by milking snakes kept at the Institute of Immunology Inc., Zagreb, Croatia. It was desiccated at room temperature and stored in the dark at 4 °C. Before this study, *Vaa* venom solution was prepared in 0.9% NaCl at a concentration of 10 mg mL^−1^, and its lethal dose was determined. “Zagreb” antivenom (batch number 190/2) was provided by the Institute of Immunology Inc., Zagreb, Croatia, and stored at 4 °C. Recombinant ammodytoxin A (AtxA) was provided by the Institute Jožef Stefan, Ljubljana, Slovenia.

### 2.2. Animals

This study was conducted on a total of 16 mixed female Pramenka sheep (weighing about 50 kg) obtained from a local family farm. The animals used for blood sampling were in the experiment for two weeks. Those used for lymph sampling were in the experiment for six hours and were closely monitored using a clinical assessment form, with more frequent checks on behavior and body weight. They were housed in enriched enclosures with sufficient floor space to allow them to lie down, chew their cud, and move freely. The entire enclosure had a solid floor covered with straw, a ventilation system with a temperature of 18–21 °C, a relative humidity of 30–50%, with a minimum of 6 h of darkness, and unrestricted access to water and food consumed ad libitum.

### 2.3. Ethical Statement

All animal experiments complied with the ARRIVE guidelines 2.0 [53] and were carried out in accordance with EU Directive 2010/63/EU on the protection of animals used for scientific purposes. Animal experimentation was approved by the Croatian Ministry of Agriculture, Veterinary and Food Safety Directorate (UP/I-322-01/20-01/62, permission no. 525-10/1338-21-5). The approval was based on the positive opinion of the National Ethical Committee (EP 315/2021).

### 2.4. Study Design

**Figure 1:**
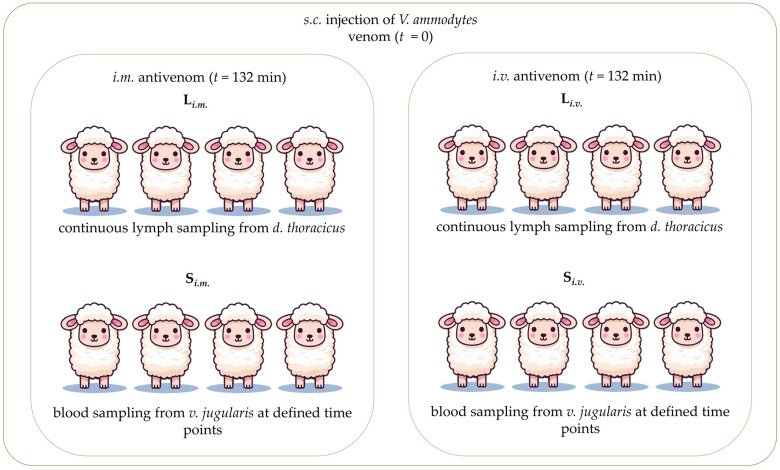
The venom-treated animals are divided into two groups, according to the route of antivenom administration. In one group (*n* = 8), antivenom is administered by *i.m.* bolus. In another group (*n* = 8), antivenom is administered by *i.v.* infusion over 30 min. In each group, half of the animals are used for lymph sampling (L*_i.m_.* and L*_i.v._* groups). The other half are used for blood sampling (S*_i.m._* and S*_i.v._* groups).

Sixteen sheep were given a 20 mg dose of venom, applied *s.c.* They were divided into two groups, according to the route of antivenom administration (Figure 1). Group 1 (*n* = 8) received an *i.m.* antivenom injection (4 mL of a 100 mg mL^−1^ solution). Group 2 (*n* = 8) obtained *i.v.* antivenom as an infusion (4 mL of a 100 mg mL^−1^ solution diluted in 100 mL of 0.9% NaCl) over 30 min. In group 1, half of the animals were used for lymph sampling (L*_i.m._*) and the other half for blood sampling (S*_i.m._*). Similarly, in group 2, half of the animals were used for lymph sampling (L*_i.v._*) and the other half for blood sampling (S*_i.v._*).

### 2.5. Surgical Procedure for Lymph Sampling

Sheep were premedicated with xylazine (0.1 mg/kg *i.m.*) and ketamine (7.5 mg/kg *i.m.*). After placement of the *i.v.* catheter, fentanyl (2 μg/kg *i.v.*) and diazepam (0.25 mg/kg *i.v.*) were administered, and anesthesia was induced with thiopental (3.1 mg/kg *i.v.*). Sheep were then intubated with an endotracheal tube (10 mm internal diameter) during the surgical phase of anesthesia. Intraoperative anesthesia was maintained with the inhalational anesthetic isoflurane (1.5–2%), and continuous infusion of the opioid analgesic fentanyl (0.1–0.2 µg/kg/min *i.v.*) provided additional analgesia. Lactated Ringer’s solution was administered *i.v.* during the operation at a rate of 10 mL/kg/min. Vital signs (ECG, pulse oximetry, capnography, arterial blood pressure) were monitored, and an active warming method was applied throughout the procedure. The antibiotic cefazolin (22 mg/kg *i.v.*) was applied prophylactically every 90 min during surgery only. An intercostal thoracotomy was performed in the eighth intercostal space on the right side. A methylene blue solution was applied to the left hind leg to visualize the thoracic duct and to begin lymph aspiration over the next 4–6 h. The volume of the lymph collected was replaced with 0.9% NaCl. At the end of the procedure, the thoracic duct was ligated, and the intercostal thoracotomy incision was closed. The health status of the animals was monitored at 6 h intervals post-operatively, and additional analgesia was administered as required. It was provided by a fentanyl patch (75 μg/kg *s.c.*) and the use of the non-steroidal anti-inflammatory drug meloxicam (0.1 mg/kg *s.c.*) for 5 days. A standardized pain scale was used to assess the severity of pain, suffering or distress.

### 2.6. Venom and Antivenom Application

*V. ammodytes* venom solution was administered *s.c.* into the interdigital space of the left hind limb of each sheep. Venom was injected at a dose of 20 mg, which is a typical quantity yielded in milking of a mature snake and contained 1849 LD_50_ doses as determined by the lethal toxicity assay [54]. “Zagreb” antivenom was administered 132 min after venom-induced envenoming at a dose of 400 mg, which was found to be sufficient to neutralize 1947 LD_50_ doses of *V. ammodytes* venom as determined by the lethal toxicity neutralization assay.

### 2.7. Lymph Samples

**Figure 2:**
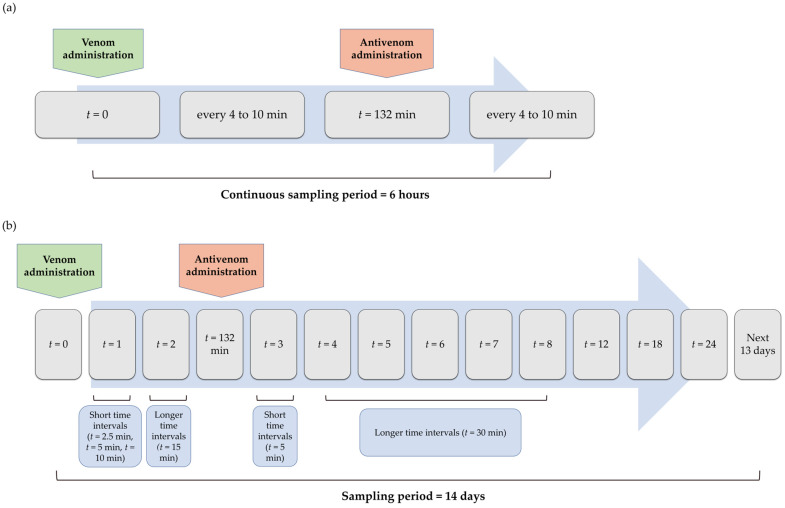
Protocol for lymph (**a**) and blood sampling (**b**) in envenomed and antivenom-treated sheep.

Lymph samples were collected by external drainage from the thoracic duct after surgery. Lymph was continuously aspirated over 4–6 h into separate 15 mL tubes that were changed every 4–10 min, depending on the flow rate (Figure 2a). After volume estimation, lymph samples were centrifuged to remove clots, aliquoted, and stored at −20 °C until venom, ammodytoxins (Atxs), and antivenom were measured. The lymphatic absorption of venom, Atxs and *i.m.* and *i.v.* antivenoms was monitored by measuring their concentrations in continuously collected lymph samples and expressed as cumulative absorbed doses. In each sample, it was obtained by summing the values of all previous intervals.

### 2.8. Blood Samples

For the pharmacokinetic study, blood samples were collected from the jugular vein into 8.5 mL serum tubes at defined time points after the insertion of an intravenous catheter (20G) and fixation with two sutures (Dafilon 3-0). After venom administration, blood was collected at closely spaced intervals for the first hour, extended to 15 min until antivenom administration at 132 min, after which the sampling intervals decreased and became progressively longer over the next 6 h (Figure 2b). Samples were also collected at 12, 18 and 24 h after venom application and daily for the next 13 days. The last sample collected was used for the evaluation of anti-antivenom antibodies. Centrifuged samples were frozen at −20 °C until concentrations were measured. To monitor venom-induced blood disorders, blood samples of L and S groups were collected from the jugular vein in EDTA/sodium citrate tubes immediately before envenomation and at 0.5, 1, 1.5, and 2 h after envenomation, then hourly for the next 5 h, and daily for one week. They were analyzed straight away.

### 2.9. Quantification of V. ammodytes Venom in Lymph and Serum Samples

Venom concentrations were determined by an in-house sandwich ELISA assay by coating a microtiter plate with a rabbit antivenom IgG solution (100 µL/well of 5 µg mL^−1^) in a 0.05 M carbonate buffer, pH 9.6. After overnight incubation at room temperature (RT), the plate was washed and blocked with the blocking buffer (2% (*m*/*v*) solution of bovine serum albumin (BSA) in a PBS buffer containing 0.05% (*v*/*v*) Tween 20) (250 µL/well) for 4 h at 37 °C. Lymph and serum samples were prepared in the incubation buffer (0.5% (*m*/*v*) BSA solution in a PBS buffer containing 0.05% (*v*/*v*) Tween 20) according to the expected values of their absorbances. The venom solution (*c* = 1 mg mL^−1^), used as the standard, and its eight serial 1.5-fold dilutions starting from 15 ng mL^−1^ were prepared in an incubation buffer with the respective percentage (*v*/*v*) of the lymph/serum (collected prior to venom administration). All samples and standards, as well as negative controls (lymph/serum collected prior to venom administration) were added in duplicates (100 μL/well). After overnight incubation at RT and a thorough washing, the plate was incubated with an anti-*V. ammodytes* venom IgG solution (100 µL/well of 5.7 µg mL^−1^) and then with a rabbit HRP-anti-equine IgG solution (100 µL/well of 4000-fold dilution). Each incubation was performed for 2 h at 37 °C. Washing was followed by a half-hour incubation step with an *o*-phenylenediamine (OPD) solution (0.6 mg mL^−1^) in a 5.5 mM citrate-phosphate buffer, pH 5.0, with 30% (*v*/*v*) H_2_O_2_ (0.5 µg mL^−1^ of OPD solution) (100 µL/well) at RT in the dark. After the addition of a 12.5% (*v*/*v*) H_2_SO_4_ solution (50 µL/well), the absorbance was measured at 492 nm. To calculate the venom content, the corresponding dilution factor was multiplied by each concentration that was determined from the standard curve. The cut-off value was the mean absorbance of the lymph/serum sample collected prior to venom administration, which was analyzed in the lowest dilution used for venom-containing samples, plus three standard deviations. All samples for which the absorbance obtained in the assay was less than the cut-off value were assigned a concentration of 0 ng mL^−1^.

### 2.10. Quantification of Atxs in Lymph and Serum Samples

Atx concentrations were determined by an in-house sandwich ELISA assay, following the same protocol as for venom, with some variations. A microtiter plate was coated with a rabbit anti-Atx IgG solution (100 μL/well of 5 µg mL^−1^). Lymph and serum samples were prepared and analyzed in the same manner as for venom. The AtxA solution (*c* = 10 µg mL^−1^) was used as the standard, and its eight serial 1.5-fold dilutions starting from 1.5 ng mL^−1^ were prepared in the appropriate matrix. After incubation and washing, the plate was incubated with a guinea pig anti-AtxA IgG solution (100 µL/well of 1.5 µg mL^−1^) followed by a rabbit HRP-anti-guinea pig IgG solution (100 µL/well of 10,000-fold dilution). The final steps and calculations were performed as described in Section 2.9.

### 2.11. Quantification of Antivenom in Lymph and Serum Samples

Concentrations of the antivenom (i.e., venom-specific F(ab’)_2_ fragments) were determined by the in-house direct ELISA assay by coating a microtiter plate with *V. ammodytes* venom solution (100 µL/well of 1 µg mL^−1^) in a 0.05 M carbonate buffer, pH 9.6. After overnight incubation at RT, the plate was washed and blocked with the blocking buffer for 2 h at 37 °C. Lymph and serum samples were prepared in the incubation buffer according to the expected values of their absorbances and added in duplicates (100 µL/well) in five serial 2-fold dilutions. “Zagreb” antivenom used as the standard was added in duplicates (100 μL/well) in eight serial 2-fold dilutions starting from 200 ng mL^−1^. Dilutions were prepared in the incubation buffer. After overnight incubation at RT and a thorough washing, the plate was incubated with goat anti-horse F(ab’)_2_ IgG-HRP (100 µL/well of 5000-fold dilution) for 2 h at 37 °C. The final steps and calculations were performed as described in Section 2.9.

### 2.12. Quantification of IgGs Specific for the Antivenom’s F(ab’)_2_ Fragments in Serum Samples

Concentrations of IgGs specific for the antivenom’s F(ab’)_2_ fragments were determined by the in-house direct ELISA assay by coating a microtiter plate with an antivenom solution (100 µL/well of 1 µg mL^−1^) in a 0.05 M carbonate buffer, pH 9.6. After overnight incubation at RT, the plate was washed and blocked with the blocking buffer for 2 h at 37 °C. Serum samples were prepared in the incubation buffer according to the expected values of their absorbances and added in duplicates (100 µL/well) in serial 2-fold dilutions. After overnight incubation at RT and a thorough washing, the plate was incubated with rabbit anti-ovine IgG-HRP (100 µL/well of 10,000-fold dilution) for 2 h at 37 °C. The final steps were performed as described in Section 2.9. Anti-antivenom antibodies were quantified by a parallel line assay comparing each sample to an “in-house standard”, a serum containing high levels of IgGs specific for the antivenom’s F(ab’)_2_ fragments, to which 100 arbitrary units per mL (AU mL^−1^) were assigned.

### 2.13. Hemostasis and Coagulation Tests

Platelets, leukocytes, and erythrocytes were assayed using the CELL-DYN Ruby Hematology Analyzer (Abbott, IL, USA). Platelet aggregation was measured on a Multiplate Analyzer (Roche, Mannheim, Germany). Hemostasis testing was performed using the BCS XP System (Siemens Healthineers, Erlangen, Germany). For each animal, the results were expressed as the percentage of deviation from the value estimated before venom application.

### 2.14. Data Analysis

Each assay was independently repeated at least three times, and the average result was used for all further calculations. The pharmacokinetic analysis of measured concentrations was performed using PKSolver add-in software (version 2.0, China Pharmaceutical University, Nanjing, China) for Microsoft Excel [55]. The noncompartmental model after extravascular or intravenous constant infusion input was selected to calculate the elimination half-life (*t*_1/2_), time to reach maximum plasma concentration (*t*_max_), maximum plasma concentration (*c*_max_), apparent volume of distribution during terminal phase (*V*_z_), mean residence time (MRT), area under the plasma concentration–time curve (AUC) and apparent total body clearance (CL). Results were expressed as the mean *±* standard error (SE) or median (range between lower and upper percentiles), unless otherwise noted. The area under the lymph mass–time curve was determined using Prism software (version 9.0, GraphPad Software Inc., San Diego, CA, USA). Comparisons between the *i.m.* and *i.v.* groups were evaluated by U-test at a significance level of *p* < 0.05, also using Prism software.

## 3. Results

### 3.1. Lymphatic System

#### 3.1.1. Antivenom

**Figure 3:**
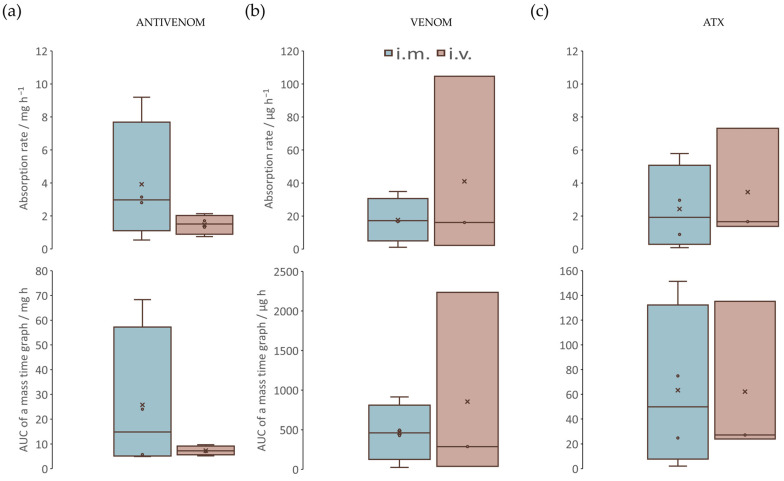
The lymphatic absorption in envenomed and antivenom-treated sheep. The rate of absorption and the total amount reaching the lymph during a sampling period (AUC_0-*t*_) of *i.m.* and *i.v.* antivenoms (**a**), as well as of the venom (**b**) and ammodytoxins (Atxs), in relation to the route of antivenom administration (**c**). Comparisons between the L*_i.m._* group, treated with *i.m.* antivenom, and the L*_i.v._* group, treated with *i.v.* antivenom, are evaluated by U-test at a significance level of *p* < 0.05.

The lymphatic absorption of *i.m.* and *i.v.* antivenoms was monitored by measuring their concentrations (Tables S1 and S2). When comparing the L*_i.m._* and L*_i.v._* groups, both the rate of absorption of *i.m.* antivenom (3.9 ± 1.9 mg h^−1^) and its total amount reaching the lymph during a sampling period (AUC_0-*t*_) (25.7 ± 14.9 mg ∙ h) appeared to be generally higher than for *i.v.* antivenom (Figure 3a, Table S3). The rate of absorption of *i.v.* antivenom was 1.5 ± 0.3 mg h^−1^. Its AUC_0-*t*_ was 7.3 ± 1.1 mg ∙ h. Overall, the L*_i.m._* group appeared to be exposed to a faster arrival of a larger dose of *i.m.* antivenom over time. However, within the L*_i.m._* group, a high variability in its absorption was observed. On the contrary, within the L*_i.v._* group, a more consistent level of *i.v.* antivenom exposure was ensured. As such, none of the differences appeared to be significant (*p* > 0.05), both in terms of rate and total dose.

#### 3.1.2. Venom and Atxs

**Figure 4:**
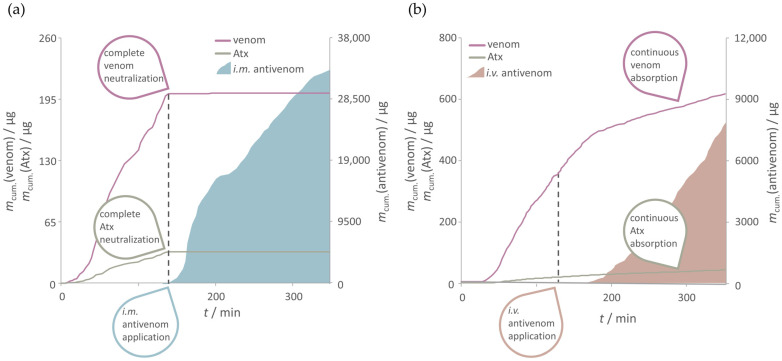
Representative example of the cumulative increase in the venom, ammodytoxins (Atxs) and antivenom quantities absorbed into the lymphatic circulation after the *s.c.* injection of venom and *i.m.* (**a**) or *i.v.* injection of antivenom (**b**).

The lymphatic absorption of either the whole venom or ammodytoxins (Atxs) was monitored by measuring their concentrations (Tables S1 and S2). In the L*_i.m._* group, *i.m.* antivenom, even at low doses (about 500 µg on average), rapidly (after an average of 23 min) succeeded in complete neutralization of the venom present in the lymphatic circulation (Figure 4a). Atxs were completely neutralized somewhat earlier (after an average of 15 min), when *i.m.* antivenom was absorbed at an average dose of only 13 µg. On the contrary, in the L*_i.v._* group, *i.v.* antivenom-mediated neutralization of the venom and Atxs was only partial (Figure 4b). The rate of venom absorption (17.6 ± 6.9 µg h^−1^ vs. 41.0 ± 32.1 µg h^−1^) and the total amount of venom reaching the lymph during the sampling period (AUC_0-*t*_) (465.6.6 ± 256.6 µg ∙ h vs. 853.8 ± 694.6 µg ∙ h) were generally lower when the antivenom was given *i.m.* vs. *i.v.*, respectively (Figure 3b, Table S3). Due to the high variability in the venom exposure levels within the L*_i.v._* group, the differences could not be proved as significant (*p* > 0.05) for any of the observed parameters. In contrast to venom, the absorption rate of Atxs and their AUC_0-*t*_ were congruent in both groups, regardless of the administration route (Figure 3c, Table S3).

### 3.2. Systemic Circulation

#### 3.2.1. Antivenom

**Figure 5:**
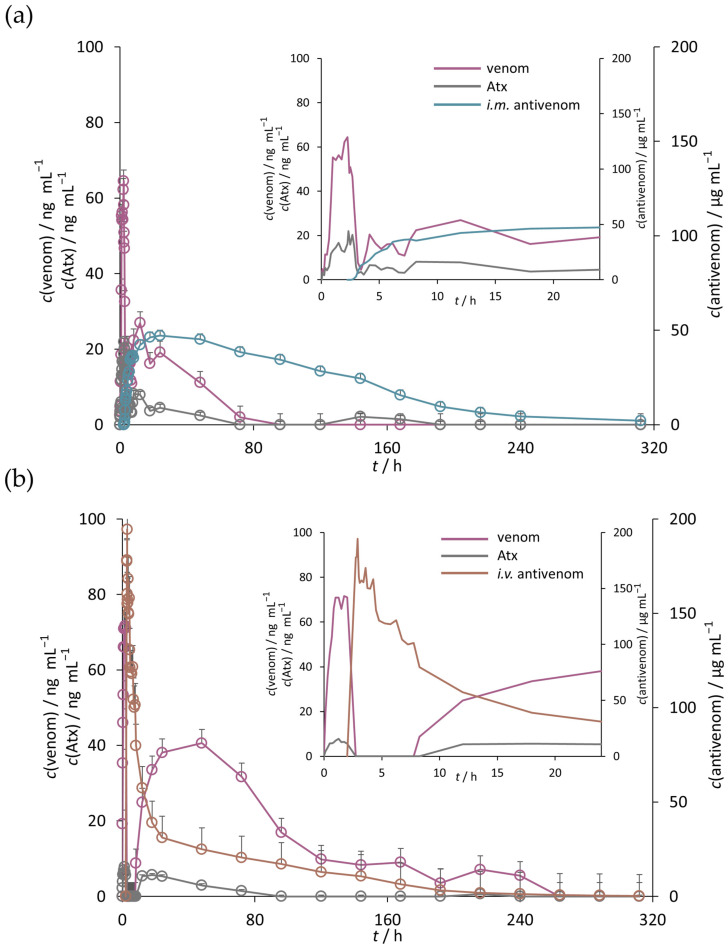
Venom and antivenom pharmacokinetics in the systemic circulation. Representative pharmacokinetic profiles of venom, ammodytoxins (Atxs) and *i.m.* administered antivenom in the systemic circulation of a sheep from the S*_i.m._* group during the entire sampling period and during the first 24 h (excerpt) (**a**). Representative pharmacokinetic profiles of venom, Atxs and *i.v.* administered antivenom in the systemic circulation of a sheep from the S*_i.v._* group during the entires ampling period and during the first 24 h (excerpt) (**b**). Results are presented as the mean of three independent measurements ± standard error.

**Figure 6:**
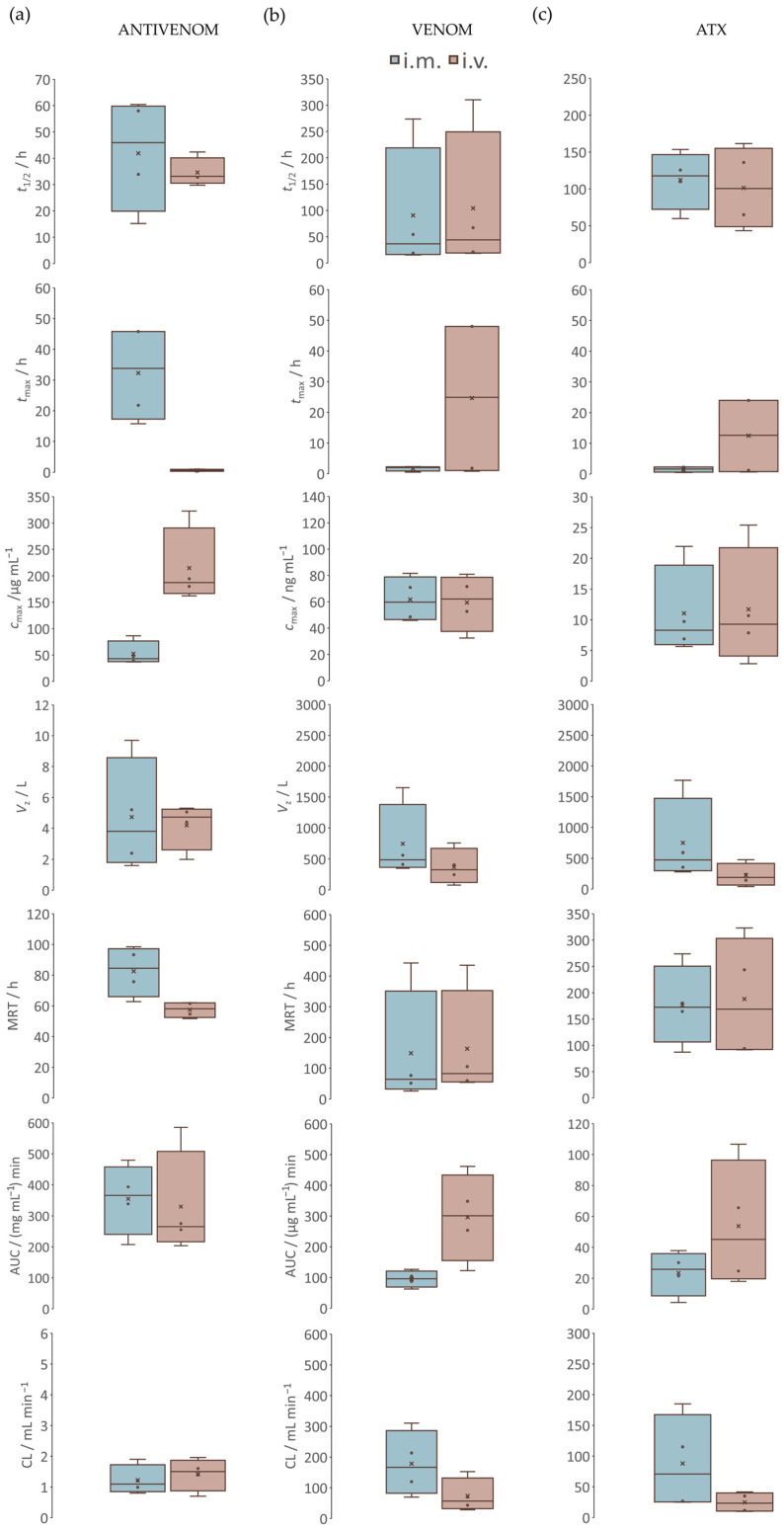
Pharmacokinetic parameters in the sera of envenomed and antivenom-treated sheep. The elimination half-life (*t*_1/2_), time to reach maximum concentration (*t*_max_) and maximum concentration (*c*_max_), volume of distribution (*V*_z_), mean residence time (MRT), total amount in the bloodstream over the duration of the measurement period (AUC_0-*t*_) and clearance (CL) of antivenoms (**a**), as well as of the venom (**b**) and ammodytoxins (Atxs) (**c**), in relation to the route of antivenom administration, are presented. Comparisons between the S*_i.m._* group, treated with *i.m.* antivenom, and the S*_i.v._* group, treated with *i.v.* antivenom, are evaluated by U-test at a significance level of *p* < 0.05.

Antivenom concentrations (Figure 5, Tables S4 and S5) were used for the determination of pharmacokinetic parameters (Figure 6a, Table S6). In the systemic circulation of the S*_i.m_*_._ group, the maximum concentration (*c*_max_) of antivenom was 52.3 ± 11.6 µg mL^−1^. In the systemic circulation of the S*_i.v_*_._ group, the *c*_max_ of antivenom was 214.8 ± 36.6 µg mL^−1^. The time required to reach it (*t*_max_) in the S*_i.m._* group was 32.3 ± 7.9 h. In the S*_i.v_*_._ group, the *t*_max_ was 0.6 ± 0.2 h. Differences in the *c*_max_ and *t*_max_ proved significant. Both approaches resulted in a similar elimination half-life of the antivenom (*t*_1/2_), as well as the total amount in the bloodstream over the duration of the measurement period (AUC_0-*t*_), the volume of distribution (*V*_z_), and clearance (CL). However, the mean residence time (MRT) was significantly longer after *i.m.* administration (82.6 ± 8.2 h) than after *i.v.* administration (57.5 ± 2.6 h).

#### 3.2.2. Venom and Atxs

The concentrations of venom and Atxs (Figure 5, Tables S4 and S5) were used for the determination of pharmacokinetic parameters (Figure 6b,c, Table S6). In both groups, the maximum venom concentrations (*c*_max_) measured in serum were congruent but reached at different times (Table S6). In the S*_i.m_*_._ group, the *c*_max_ was achieved either during the pre-treatment period or immediately after antivenom administration and just before neutralization of the venom, manifested by an immediate drop in its concentration to baseline (Figure 5a). Complete neutralization occurred 4 days post-antivenom administration, with the exception of one animal in which only two hours were required. In the S*_i.v._* group, the *c*_max_ of venom was achieved either during the pre-treatment period or even 2 days after antivenom administration. Namely, the initial neutralization of the venom, which occurred a few minutes post-antivenom administration, was followed by a pronounced secondary rise in its serum concentrations (Figure 5b). The phenomenon was also observed in the S*_i.m._* group but to a lesser extent (Figure 5a). Namely, total venom exposure during the post-treatment period was three times lower when antivenom was administered *i.m.* in comparison to that given *i.v.* (93.3 ((µg mL^−1^) ∙ min) vs. 285.0 ((µg mL^−1^) ∙ min). In addition, *i.m.* antivenom provided a higher volume of distribution (*V*_z_) and clearance (CL) of the venom (Figure 6b, Table S6). Congruent results were obtained when the pharmacokinetics of Atxs were followed (Figure 6c, Table S6).

### 3.3. Hematological and Coagulation Parameters

**Figure 7:**
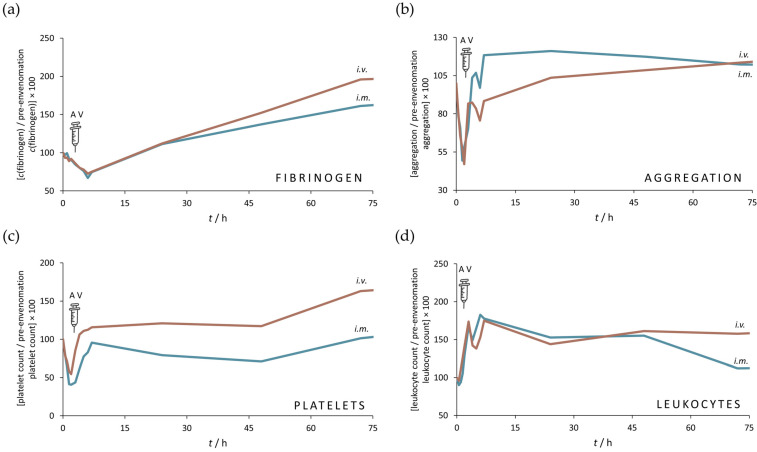
Impact of *i.m.* and *i.v.* antivenoms on the venom-induced blood disorders. Fibrinogen concentration (**a**), degree of aggregation (**b**), platelet (**c**) and leukocyte count (**d**) are determined in blood samples taken before and after envenoming and during the post-treatment period. The results are given as the mean of the values obtained for each animal.

Envenoming caused a marked decrease in fibrinogen concentration, along with a reduction in platelet count and aggregation ability. The most pronounced drop in fibrinogen concentration, approximately 70% from baseline, was measured 6 h post- envenomation, while the onset of recovery was evident 5 h after antivenom treatment (Figure 7a). Similarly, platelet aggregation showed a substantial decline of about 50% from baseline, measured 1.5 to 2 h post-envenomation, with improvement observed within 1 h after antivenom administration (Figure 7b). The most pronounced drop in platelet count, aproximately 50% from baseline, was measured 2 h post-envenomation, while the onset of recovery was evident 1 h after antivenom treatment (Figure 7c). The time from antivenom administration to the recovery start was independent of the administration route. Also, envenoming was accompanied by ongoing leukocytosis, except for a transient decrease that occurred 4 h after envenomation (Figure 7d). Antivenom administration did not affect the increase in leukocyte count, regardless of the therapeutic principle. *V. ammodytes* venom had no effect on prothrombin time, activated partial thromboplastin time, D-dimer levels and red blood cell count, which remained within the physiological range throughout the entire measurement period.

### 3.4. Humoral Response Against Equine F(ab’)_2_ Fragments

**Figure 8:**
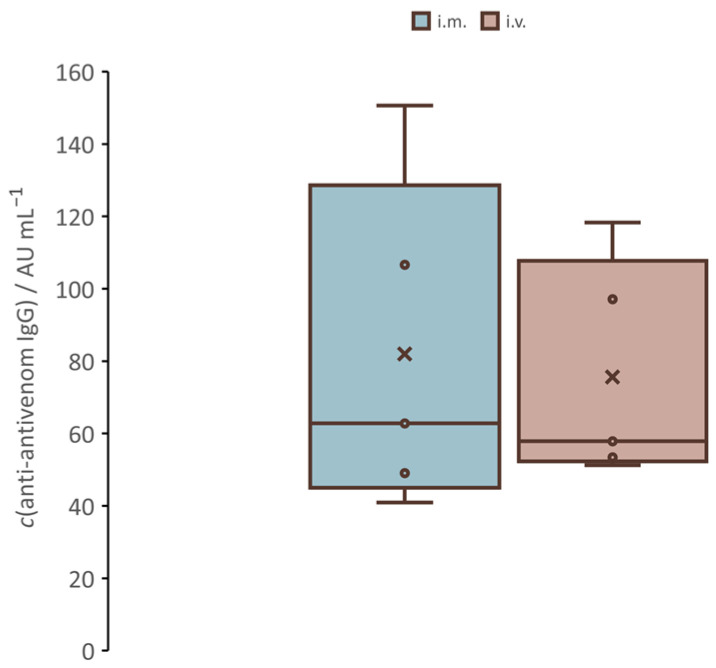
Humoral response to *i.m.* and *i.v.* antivenoms. Concentrations of anti-antivenom IgGs in serum samples taken 2 weeks after antivenom administration are determined by ELISA and expressed in arbitrary units (AUs) per mL. The comparison between the S*_i.m._* group, treated with *i.m.* antivenom, and the S*_i.v._* group, treated with *i.v.* antivenom, is evaluated by U-test at a significance level of *p* < 0.05.

The evaluation of IgGs raised against equine F(ab’)_2_ fragments in serum samples of the animals from the rescue study, which were collected two weeks after therapeutic intervention, was assesed. There was no significant difference in the humoral response between animals that received antivenom by the *i.m.* route and those that received it by the *i.v.* route (U test, *p* > 0.05) (Figure 8).

## 4. Discussion

The majority of previous experimental investigations that attempted to clarify the pharmacokinetics of antibody-based therapeutics, including antivenoms, either alone or in combination with the corresponding venom, have been restricted to concentration level monitoring in the systemic circulation [21,22,23,24]. It has only recently become clear that the lymphatic system cannot be ignored in a proper understanding of venom/antivenom interactions, given its important role in the transport of interstitial fluid and extravasated proteins [44,46,56]. No other study, prior to the one described here, has attempted to elucidate the pros and cons of *i.m.* and *i.v.* antivenoms in a comprehensive and comparative manner, taking into account their neutralization efficacy in both relevant body compartments. In addition to filling in the missing gaps, the ability of *i.m.* and *i.v.* antivenoms to reverse blood disorders and their safety profiles were investigated, all with the aim of identifying the optimal course of action for envenoming therapy.

The absorption of *i.m.* and *i.v.* antivenoms into the lymphatic system was demonstrated, but differences were observed in the rate of absorption, the total amount reaching the lymph, and the ability to neutralize the circulating venom. The *i.m.* antivenom appeared to behave more favorably. Due to its better absorption properties, it was more successful in slowing down the arrival of the venom and, consequently, reducing the total amount reaching the lymph. More importantly, only *i.m.* antivenom ensured the complete neutralization of the lymph-absorbed venom. In another study in which antivenom was administered by the *i.v.* route, unbound venom also remained detectable in the lymph until the end of the experiment [48]. The authors explained this by the fact that the *i.v.* antivenom concentrations in the lymph were lower than in the serum, having reached it as a result of extravasation from the blood, whereas the venom concentrations in the lymph exceeded those in the serum as a consequence of the absorption from the injection site, which acts as a depot for the extended delivery. So, the lymph appears to serve as a slow release compartment, prolonging venom exposure and compromising its neutralization by the *i.v.* antivenom, whose delayed entry from the blood may be a key limiting factor in reducing the systemic availability of the toxic components [45]. This is consistent with our observation that the venom persisted in the lymph even after administration of the *i.v.* antivenom, emphasizing the importance of its interrupted absorption achieved with the *i.m.* antivenom. Our findings highlight the pronounced lymphatic mismatch between venom and *i.v.* antivenom, with *i.m.* administration providing more efficient venom clearance from the lymph.

The number of studies directly comparing the effects of *i.v.* and *i.m.* antivenoms on the pharmacokinetic profile of the venom in the systemic circulation is limited. Furthermore, differences in venom/antivenom interplay observed not only between animals and humans but also between animal models chosen according to the focus of the research and the type of snake venom [57,58] make it difficult to draw straightforward conclusions. All this impairs a comprehensive understanding of the venom behavior associated with different routes of antivenom administration, which can be a significant drawback in optimizing the therapeutic protocol. Our study aimed to provide all possible data that could be calculated using non-compartmental analysis. The consistency of the elimination half-life of *i.m.* and *i.v.* antivenoms was demonstrated, confirming previous results [21,23]. However, *i.m.* antivenom appears to result in a higher volume of distribution of the venom, indicating a greater propensity for toxins to leave the systemic circulation and enter the extravascular compartment, potentially reducing the efficacy of the drug [59], as it struggles to neutralize target components already distributed in the surrounding tissues [60]. On the other hand, *i.m.* antivenom also appears to provide a higher clearance of the venom, as another important pharmacokinetic parameter representing the efficiency of the irreversible elimination of a drug. It is important to note that both the volume of distribution and clearance are correlated and together influence the elimination half-life of the venom [59]. A larger volume of distribution will typically result in a longer elimination half-life by sequestering the drug in tissues away from the bloodstream. On the other hand, a higher clearance rate accelerates its removal from the systemic circulation, thereby shortening the elimination half-life. As shown in our study, these opposing effects may cancel each other out so that the venom with a higher volume of distribution and clearance, such as after *i.m.* administration of the antivenom, may have an elimination half-life comparable to that of the venom with a lower volume of distribution and clearance, such as after *i.v.* administration of the antivenom. Therefore, it is the interaction between these two parameters, rather than their individual values, that is critical in assessing the elimination half-life. The total amount of antivenom in the bloodstream during the measurement period appeared to be comparable for both administration routes. Although our finding is not in agreement with previous ones [21,23,30], it can be explained by the slow, but sustained release of *i.m.* F(ab’)_2_ fragments from the injection site [61,62], leading to drug accumulation and resulting in similar systemic exposure as after *i.v.* administration. A sufficiently long measurement time may be required for the effect to appear. Furthermore, pharmacokinetic differences were observed in the maximum plasma concentration and the time required to reach it. When the antivenom was administered by the *i.m.* route, the *c*_max_ was significantly lower, and the *t*_max_ was significantly longer, extending to 1.3 days, which is in line with other reports showing that it can occur even up to two days post-treatment [21,22,23,62]. Described delay has been attributed to the prolonged arrival of the antivenom from the muscle tissue [40,62]. Despite the slower absorption, *i.m.* antivenom offers an advantage in situations where a sustained release and prolonged maintenance of its levels is required, such as in the treatment of viper envenomation, where the initial entry of the toxins from the *s.c.* tissue surrounding the bite site is followed by their continuous release for up to 3 days [29]. As *i.m.* antivenom provides ongoing protection, recurrent envenomation is likely to be less common [63], which is also supported by our results. Specifically, the reappearance of the venom in the systemic circulation was much less pronounced when antivenom was administered by the *i.m.* route compared to the *i.v.* route, as clearly indicated by the three times lower total exposure measured during the post-treatment period. This may be due to the presence of a venom reservoir in the local tissues and lymph, whose flux exceeds the binding capacity of the *i.v.* antivenom as it begins to be cleared from the systemic circulation. *I.m.* administration proved effective in maintaining lower blood venom levels over time, most likely due to the steady increase in antivenom concentrations [64]. *I.v.* administration resulted in a rapid drop in venom blood levels, which may be useful in the cases of acute envenomation. However, the effect was transient, and there was a marked rebound in venom concentrations, probably due to re-circulation of the toxins stored in the peripheral tissues or the lymphatic system into the bloodstream [65] or due to premature elimination of the antivenom [40]. The beneficial effect of the *i.m.* antivenom on the removal of the venom from the systemic circulation may be supported by its longer mean residence time, a phenomenon already reported [62,66], as it allows a prolonged therapeutic effect of the drug due to the circulation through organ tissues [28].

The immune system requires 1–2 weeks after antivenom administration to recognize the heterologous equine antibodies as foreign and to mount an IgG-mediated antibody response that can lead to late adverse reactions, such as a type III hypersensitivity phenomenon associated with serum sickness that appears as a consequence of the formation of immune complexes [67,68]. However, the presence of anti-antivenom antibodies is not sufficient for the late adverse reactions to occur [69]. The incidence of serum sickness seems to depend on the total load of foreign protein administered and the format of the antivenom preparation [70]. However, as the low immunogenicity of the drug is preferable to improve product safety, our goal was to investigate the effect of administration routes on the humoral response to equine F(ab’)_2_ fragments between animals receiving *i.m.* antivenom and those receiving *i.v.* antivenom. No significant difference was observed, as described [71]; thus, neither route was favored from a safety perspective. This can be explained by the overall low immunogenicity of F(ab’)_2_ fragments, which provides a solid basis for understanding why the route of administration does not significantly alter the immune response when using this type of antibody format. However, the initial evaluation is not sufficient for a definitive comparison, particularly when applied to other, more immunoreactive antivenoms. The slow release of *i.m.* antivenom may have additional safety implications that should be studied in more detail to understand the wider application.

All sheep exhibited clear evidence of envenomation almost immediately after injection of the venom. Hypofibrinogenemia, thrombocytopenia associated with reduced platelet aggregation and leukocytosis developed progressively over time. Congruent clinical signs and symptoms were often observed in human victims of the *V. ammodytes* bite [12,40,72,73,74,75,76,77]. The antivenom had a positive effect on the outcome, as the normal values of the laboratory results were quickly restored after its administration, confirming the therapeutic efficacy regardless of the route. Only the leukocyte count was not reversed by the antivenom, as previously reported [72,77], and continued to increase possibly due to the inflammatory process and cytokine production [78] triggered by severe local tissue damage, one of the most frequently recorded effects of *V. ammodytes* envenomation [59]. According to Hsu et al., an elevated value of this parameter may contribute to the early diagnosis of compartment syndrome [79].

## 5. Conclusions

Our study demonstrated for the first time not only the neutralizing effect of *i.m.* antivenom in the lymphatic system but also allowed us to compare the impact of *i.m.* and *i.v.* routes on venom depletion in both relevant body compartments where interaction with circulating toxins occurs. These results are in agreement with Kurtović et al. [40], who highlighted the potential of *i.m.* antivenom to provide early therapeutic levels, particularly in emergency situations, without the need for additional doses to fully resolve all clinical signs. This study has disproved the negative connotation associated with *i.m.* administration, proving that it is an equally good concept from the standpoint of removing the venom from both the lymph and blood, as well as normalizing hematologic abnormalities commonly observed in viperid envenomations. However, it should be noted that its efficacy is currently only applicable to *V. ammodytes* venom and not to snake venoms in general. Also, awareness of the limited usefulness of *i.m.* antivenom should be cautioned, especially in light of previous work in mice [28]. Future studies are needed to draw firm conclusions. Finally, the pharmacokinetic properties of different venom/antivenom combinations vary considerably, mainly due to differences in molecular size. A recent computational model provided a theoretical framework to understand their impact on the behavior of these substances in the body, as well as the efficacy and timing of treatment [80]. These pharmacokinetic differences should be considered beyond the specific venom/antivenom combination that we studied, with broader implications for other venom types and antivenom formats.

## Data Availability

The original contributions presented in this study are included in the article/Supplementary Material. Further inquiries can be directed to the corresponding author.

## Supplementary Materials

The following supporting information can be downloaded at https://www.mdpi.com/article/10.3390/pharmaceutics17020212/s1, Table S1: Concentrations of venom, Atx and antivenom measured in the lymph samples of envenomed and *i.m.*-treated sheep; Table S2: Concentrations of venom, Atx and antivenom measured in the lymph samples of envenomed and *i.v.*-treated sheep; Table S3: Lymphatic absorption of *i.m.* and *i.v.* antivenoms and its influence on the rate of absorption of *s.c.* injected venom and ammodytoxins (Atxs), as well as on their total amount reaching the lymph during a sampling period (AUC_0-*t*_); Table S4: Concentrations of venom, Atx and antivenom measured in the serum samples of envenomed and *i.m.*-treated sheep; Table S5: Concentrations of venom, Atx and antivenom measured in the serum samples of envenomed and *i.v.*-treated sheep; and Table S6: Pharmacokinetic parameters of *s.c.* applied venom (*m* = 20 mg) and ammodytoxins (Atxs), as well as *i.m.* (S*_i.m._* group) and *i.v.* (S*_i.v._* group) antivenoms (*m* = 400 mg) in the systemic circulation.
