# Supplementary material for: Pharmacokinetics of Snake Antivenom Following Intravenous and Intramuscular Administration in Envenomed Large Animal Model
Source: Pharmaceutics. 2025 Feb 7;17(2):212. doi: 10.3390/pharmaceutics17020212 (PMC11859798; doi:10.3390/pharmaceutics17020212)
Supplement: Supplementary file 1 [file pharmaceutics-17-00212-s001.zip › Supplementary Table S1.pdf]

Table S1. Concentrations of venom, Atx and antivenom measured in the lymph samples of envenomed and *i.m.*-treated sheep.

L$_{i.m.}$ – sheep 1

V / mL	*t* /min	$t_{post\text{-}AV}$ / min	*c*(venom) / ng mL^{-1}	*c*(Atx) / ng mL^{-1}	*c*(antivenom) / μg mL^{-1}
10.5	7		0.0	0.0	
12.5	12		9.1 ± 2.3	0.7 ± 0.1	
12.5	16		11.3 ± 2.0	0.9 ± 0.1	
12.5	21		27.9 ± 3.8	2.3 ± 0.3	
13.0	25		30.4 ± 3.7	2.6 ± 0.3	
12.9	29		28.2 ± 2.5	2.7 ± 0.3	
12.4	33		56.7 ± 5.2	5.1 ± 0.3	
14.0	38		66.6 ± 5.0	6.4 ± 0.3	
13.0	43		46.3 ± 2.8	4.4 ± 0.1	
14.0	49		43.3 ± 3.1	4.6 ± 0.1	
13.5	54		66.0 ± 5.0	6.2 ± 0.5	
15.0	59		1462.2 ± 172.9	68.3 ± 5.0	
12.5	65		1764.5 ± 171.2	88.0 ± 2.8	
13.2	70		1163.6 ± 137.8	54.0 ± 4.4	
13.0	78		701.2 ± 97.5	30.3 ± 3.2	
12.8	90		569.0 ± 58.7	26.0 ± 3.3	
7.0	105		408.7 ± 54.6	19.9 ± 2.1	
12.7	120		761.9 ± 88.0	35.5 ± 5.5	
9.5	132	0	636.2 ± 71.3	32.5 ± 5.8	0.0 ± 0.0
8.0	146	14	573.9 ± 69.3	27.6 ± 4.5	0.0 ± 0.0
5.4	161	29	271.6 ± 32.0	11.8 ± 2.1	0.0 ± 0.0
5.5	186	54	38.4 ± 4.1	5.8 ± 0.5	0.2 ± 0.0
5.5	205	73	19.7 ± 2.6	3.0 ± 0.3	1.7 ± 0.0
5.5	233	101	3.1 ± 1.4	0.0	7.9 ± 0.2
5.4	252	120	5.5 ± 1.8	0.0	9.1 ± 0.2
5.0	272	140	3.0 ± 1.5	0.0	23.8 ± 0.3
6.4	285	153	0.0	0.0	48.4 ± 0.8
9.0	297	165	0.0	0.0	15.2 ± 0.2
10.0	302	170	0.0	0.0	27.1 ± 0.3
11.0	307	175	0.0	0.0	33.6 ± 1.5
12.3	309	177	0.0	0.0	37.6 ± 1.0
13.4	314	182	0.0	0.0	53.8 ± 2.2
12.5	318	186	0.0	0.0	50.6 ± 1.1
13.5	321	189	0.0	0.0	55.3 ± 0.5
14.5	324	192	0.0	0.0	49.9 ± 1.1
12.5	328	196	0.0	0.0	56.5 ± 0.5
14.5	332	200	0.0	0.0	58.3 ± 1.1
11.9	335	203	0.0	0.0	43.8 ± 1.1
13.5	340	208	0.0	0.0	40.7 ± 0.5
13.9	342	210	0.0	0.0	44.3 ± 1.1
15.0	346	214	0.0	0.0	44.2 ± 0.5
14.5	349	217	0.0	0.0	57.2 ± 3.0
12.5	351	219	0.0	0.0	46.5 ± 1.3
11.8	353	221	0.0	0.0	44.0 ± 1.7
12.8	355	223	0.0	0.0	51.0 ± 1.3
15.0	358	226	0.0	0.0	50.6 ± 1.9

L$_{i.m.}$ – sheep 2

V / mL	t / min	$t_{post\text{-}AV}$ / min	c(venom) / ng mL^{-1}	c(Atx) / ng mL^{-1}	c(antivenom) / µg mL^{-1}
11.5	3		0.0 ± 0.0	0.0 ± 0.0	
12.0	7		0.0 ± 0.0	0.0 ± 0.0	
12.5	10		0.0 ± 0.0	0.0 ± 0.0	
10.5	14		0.0 ± 0.0	0.0 ± 0.0	
10.5	17		0.0 ± 0.0	0.0 ± 0.0	
10.0	20		0.1 ± 0.1	0.0 ± 0.0	
11.5	24		0.2 ± 0.2	0.0 ± 0.0	
12.0	28		1.6 ± 0.9	0.0 ± 0.0	
12.5	33		0.6 ± 0.3	0.0 ± 0.0	
10.5	38		1.1 ± 0.5	0.1 ± 0.1	
13.5	43		1.4 ± 0.6	0.2 ± 0.1	
11.5	46		2.5 ± 0.7	0.2 ± 0.1	
10.0	49		1.3 ± 0.6	0.0 ± 0.0	
11.5	53		1.4 ± 0.7	0.1 ± 0.1	
13.0	58		1.8 ± 0.7	0.2 ± 0.1	
10.5	61		4.0 ± 1.0	0.1 ± 0.1	
12.5	67		2.5 ± 1.1	0.1 ± 0.1	
13.0	71		2.0 ± 0.8	0.2 ± 0.1	
7.0	78		2.6 ± 0.4	0.2 ± 0.1	
12.0	82		4.1 ± 1.0	0.2 ± 0.1	
10.5	86		2.2 ± 1.1	0.0 ± 0.0	
12.0	90		3.3 ± 0.7	0.1 ± 0.1	
12.0	95		6.8 ± 0.3	0.2 ± 0.1	
14.0	101		21.2 ± 2.9	0.4 ± 0.1	
9.5	108		42.8 ± 1.2	1.0 ± 0.1	
10.0	113		61.7 ± 3.9	3.0 ± 0.2	
14.0	119		70.8 ± 3.4	5.2 ± 0.3	
11.5	125		103.7 ± 6.4	5.8 ± 0.3	
12.5	132	0	100.6 ± 7.1	8.4 ± 0.6	0.0 ± 0.0
7.5	138	6	105.0 ± 9.2	8.6 ± 0.5	0.0 ± 0.0
8.0	142	10	38.9 ± 1.3	10.2 ± 0.7	5.6 ± 0.5
9.0	148	16	0.0 ± 0.0	3.8 ± 0.3	92.8 ± 9.6
9.5	153	21	0.0 ± 0.0	0.0 ± 0.0	103.2 ± 12.6
8.0	158	26	0.0 ± 0.0	0.0 ± 0.0	70.1 ± 7.2
8.0	164	32	0.0 ± 0.0	0.0 ± 0.0	55.0 ± 2.9
8.5	170	38	0.0 ± 0.0	0.0 ± 0.0	55.7 ± 6.3
8.5	175	43	0.1 ± 0.1	0.0 ± 0.0	64.5 ± 7.0
9.0	186	54	0.0 ± 0.0	0.0 ± 0.0	53.4 ± 5.4
8.0	195	63	0.0 ± 0.0	0.0 ± 0.0	55.3 ± 3.4
9.5	201	69	0.0 ± 0.0	0.0 ± 0.0	49.9 ± 0.6
7.5	207	75	2.2 ± 0.5	0.0 ± 0.0	33.6 ± 0.9
13.5	223	91	2.3 ± 0.4	0.0 ± 0.0	32.3 ± 0.8
10.0	234	102	1.0 ± 0.4	0.0 ± 0.0	29.1 ± 0.8
12.5	249	117	0.0 ± 0.0	0.0 ± 0.0	25.5 ± 1.8
11.5	251	119	0.0 ± 0.0	0.0 ± 0.0	18.0 ± 1.4
11.3	256	124	0.0 ± 0.0	0.0 ± 0.0	25.2 ± 0.9
14.5	263	131	0.0 ± 0.0	0.0 ± 0.0	13.2 ± 3.8
11.0	264	132	0.0 ± 0.0	0.1 ± 0.1	15.9 ± 1.6
15.0	268	136	0.0 ± 0.0	0.0 ± 0.0	15.0 ± 2.3
10.0	273	141	0.0 ± 0.0	0.0 ± 0.0	17.0 ± 1.7
14.0	277	145	0.0 ± 0.0	0.0 ± 0.0	17.8 ± 1.1
13.7	280	148	0.0 ± 0.0	0.0 ± 0.0	15.0 ± 1.4

7.5	286	154	0.0 ± 0.0	0.0 ± 0.0	15.9 ± 1.5
10.9	292	160	0.0 ± 0.0	0.0 ± 0.0	17.6 ± 1.7
10.5	299	167	0.0 ± 0.0	0.0 ± 0.0	19.2 ± 3.3
5.9	308	176	0.0 ± 0.0	0.0 ± 0.0	19.9 ± 0.8
15.0	312	180	0.0 ± 0.0	0.0 ± 0.0	16.1 ± 0.5
10.0	317	185	0.0 ± 0.0	0.0 ± 0.0	15.6 ± 0.7
11.5	324	192	0.0 ± 0.0	0.0 ± 0.0	16.8 ± 1.3
9.3	334	202	0.0 ± 0.0	0.0 ± 0.0	18.4 ± 2.5
9.0	341	209	0.0 ± 0.0	0.0 ± 0.0	17.7 ± 0.2
12.4	343	211	0.0 ± 0.0	0.0 ± 0.0	17.0 ± 0.6
11.0	351	219	0.0 ± 0.0	0.0 ± 0.0	23.5 ± 0.6
13.5	358	226	0.0 ± 0.0	0.0 ± 0.0	20.4 ± 2.1

L*i.m.* – sheep 3

V / mL	*t* / min	$t_{post\text{-}AV}$ / min	*c*(venom) / ng mL^{-1}	*c*(Atx) / ng mL^{-1}	*c*(antivenom) / µg mL^{-1}
8.5	5		0.0 ± 0.0	0.0 ± 0.0	
11.4	9		117.1 ± 9.9	22.7 ± 1.2	
5.2	12		162.2 ± 28.4	26.3 ± 3.3	
8.0	15		95.7 ± 18.8	20.1 ± 1.4	
9.5	17		95.4 ± 5.6	17.6 ± 0.8	
8.4	20		135.0 ± 10.8	22.9 ± 1.6	
8.5	24		517.8 ± 27.3	101.6 ± 9.5	
6.5	29		503.9 ± 37.7	92.4 ± 17.5	
10.6	32		401.0 ± 116.1	101.9 ± 14.3	
10.5	36		387.9 ± 29.9	83.8 ± 6.1	
11.5	39		490.1 ± 15.7	91.5 ± 4.9	
11.8	42		705.3 ± 89.6	113.2 ± 22.5	
11.8	45		777.7 ± 130.9	168.8 ± 14.7	
9.9	49		895.6 ± 14.4	70.3 ± 12.8	
12.5	53		940.5 ± 35.7	59.5 ± 7.1	
12.4	58		1610.1 ± 313.9	246.0 ± 48.3	
8.5	63		1284.8 ± 121.3	266.4 ± 13.0	
9.5	69		983.8 ± 57.3	175.7 ± 12.0	
9.7	74		929.7 ± 44.9	154.2 ± 17.2	
9.4	82		1302.1 ± 268.0	170.0 ± 40.8	
6.5	89		834.6 ± 133.6	158.0 ± 16.9	
9.0	95		561.0 ± 19.4	88.3 ± 4.6	
11.4	100		384.6 ± 34.6	58.6 ± 12.3	
9.9	103		826.0 ± 123.9	123.8 ± 31.1	
10.5	108		693.0 ± 135.7	135.2 ± 15.5	
10.1	113		542.1 ± 20.7	91.0 ± 9.4	
7.4	118		394.8 ± 29.0	63.7 ± 10.1	
10.6	121		569.9 ± 73.4	93.3 ± 20.7	
12.2	124		596.6 ± 122.1	126.5 ± 18.3	
9.5	127		480.2 ± 24.4	81.0 ± 9.2	
6.5	129		523.4 ± 7.7	83.0 ± 11.4	

6.3	132	0	821.0 ± 145.9	126.3 ± 26.7	0.0 ± 0.0
9.0	135	3	799.4 ± 84.7	165.8 ± 4.5	0.0 ± 0.0
6.0	139	7	404.0 ± 36.2	93.3 ± 9.8	0.1 ± 0.0
7.3	143	11	2.5 ± 1.5	0.0 ± 0.0	38.6 ± 4.8
7.0	150	18	2.5 ± 1.5	0.0 ± 0.0	72.5 ± 10.2
8.0	154	22	2.6 ± 2.6	0.0 ± 0.0	89.4 ± 10.8
10.0	158	26	1.3 ± 1.3	0.0 ± 0.0	64.5 ± 2.8
7.0	161	29	1.2 ± 1.2	0.0 ± 0.0	177.6 ± 30.6
10.3	164	32	0.0 ± 0.0	0.0 ± 0.0	283.1 ± 8.4
11.2	170	38	1.3 ± 1.3	0.0 ± 0.0	248.8 ± 8.1
7.0	175	43	1.3 ± 1.3	0.0 ± 0.0	194.6 ± 11.6
9.8	178	46	1.3 ± 1.3	0.0 ± 0.0	142.7 ± 26.6
8.4	182	50	3.2 ± 1.0	0.0 ± 0.0	112.2 ± 9.3
10.0	190	58	4.3 ± 2.8	0.0 ± 0.0	128.0 ± 0.9
8.8	195	63	2.7 ± 2.4	0.0 ± 0.0	117.2 ± 5.7
9.0	201	69	2.6 ± 2.3	0.0 ± 0.0	110.5 ± 12.4
5.0	206	74	4.3 ± 1.2	0.0 ± 0.0	91.3 ± 4.9
1.9	217	85	2.6 ± 2.5	0.0 ± 0.0	139.8 ± 2.4
7.5	227	95	1.8 ± 1.8	0.0 ± 0.0	110.6 ± 6.4
6.5	230	98	1.9 ± 1.9	0.0 ± 0.0	84.4 ± 9.2
9.5	232	100	1.2 ± 1.1	0.0 ± 0.0	37.2 ± 0.4
11.0	235	103	1.5 ± 1.5	0.0 ± 0.0	31.4 ± 1.3
10.0	238	106	1.1 ± 1.1	0.0 ± 0.0	44.1 ± 2.2
8.3	242	110	1.2 ± 1.2	0.0 ± 0.0	87.3 ± 9.4
9.8	246	114	1.2 ± 1.2	0.0 ± 0.0	93.2 ± 5.7
7.5	251	119	2.0 ± 2.0	0.0 ± 0.0	105.5 ± 2.5
5.3	257	125	1.4 ± 1.4	0.0 ± 0.0	126.8 ± 10.2
8.0	263	131	1.6 ± 1.6	0.0 ± 0.0	129.8 ± 25.7
6.2	268	136	0.0 ± 0.0	0.0 ± 0.0	79.0 ± 3.0
6.3	271	139	2.4 ± 2.4	0.0 ± 0.0	94.7 ± 5.9
6.4	275	143	1.3 ± 1.3	0.0 ± 0.0	96.8 ± 8.4
6.0	281	149	1.4 ± 1.4	0.0 ± 0.0	116.2 ± 22.1
10.3	289	157	1.8 ± 1.4	0.0 ± 0.0	109.5 ± 3.3
9.0	295	163	2.0 ± 2.0	0.0 ± 0.0	115.7 ± 3.5
9.0	302	170	1.5 ± 1.5	0.0 ± 0.0	95.4 ± 3.5
9.5	312	180	1.8 ± 1.8	0.0 ± 0.0	117.2 ± 13.4
9.5	317	185	2.0 ± 1.7	0.0 ± 0.0	74.3 ± 2.7
9.5	322	190	2.1 ± 1.9	0.0 ± 0.0	79.8 ± 2.6
7.3	327	195	1.7 ± 1.7	0.0 ± 0.0	69.6 ± 4.5
9.3	337	205	1.6 ± 1.6	0.0 ± 0.0	45.9 ± 3.0
12.1	347	215	1.6 ± 1.6	0.0 ± 0.0	43.2 ± 3.7

L*i.m.* – sheep 4

V / mL	*t* / min	$t_{post\text{-}AV}$ / min	*c*(venom) / ng mL^{-1}	*c*(Atx) / ng mL^{-1}	*c*(antivenom) / μg mL^{-1}
10.0	3		0.0 ± 0.0	0,0 ± 0.0	
6.5	6		0.0 ± 0.0	1,3 ± 0.1	
6.3	10		7.6 ± 0.4	2,9 ± 0.5	
6.4	14		23.0 ± 1.3	6,1 ± 0.6	
8.7	17		35.5 ± 2.0	8,5 ± 0.9	
9.3	21		33.2 ± 5.7	8,4 ± 1.1	
8.2	25		20.1 ± 1.7	4,6 ± 0.3	
6.4	30		13.9 ± 1.2	3,5 ± 0.5	
5.0	35		13.4 ± 1.8	3,3 ± 0.5	
7.8	43		21.6 ± 10.8	7,0 ± 0.5	
6.5	49		49.1 ± 19.3	18,4 ± 3.1	
6.8	54		787.1 ± 86.5	129,5 ± 10.9	
7.0	60		489.1 ± 10.6	130,5 ± 14.5	
5.4	65		662.9 ± 26.4	127,2 ± 6.0	
6.5	71		949.0 ± 113.6	155,9 ± 5.7	
6.8	75		993.6 ± 146.6	158,2 ± 13.6	
5.9	79		627.6 ± 17.1	115,1 ± 7.7	
6.6	82		616.6 ± 30.5	117,6 ± 6.2	
7.4	85		682.7 ± 121.9	100,1 ± 4.9	
10.6	89		838.1 ± 78.3	131,6 ± 7.9	
11.0	93		683.3 ± 29.6	106,8 ± 20.2	
6.6	98		502.4 ± 30.1	68,7 ± 4.3	
6.1	102		535.5 ± 90.6	86,6 ± 5.6	
9.7	110		691.6 ± 33.6	114,6 ± 6.7	
11.4	121		631.4 ± 23.4	134,4 ± 8.5	
10.2	123		637.8 ± 18.3	46,0 ± 12.2	
7.5	133	0	800.7 ± 98.1	134,5 ± 11.2	0.0 ± 0.0
10.0	148	15	604.0 ± 17.4	174,7 ± 3.8	642.5 ± 41.4
11.6	163	30	19.6 ± 2.8	1,7 ± 0.2	29100.3 ± 706.2
10.2	168	35	13.4 ± 1.5	1,0 ± 0.1	54547.8 ± 8526.7
6.4	174	41	32.0 ± 1.8	8,5 ± 0.9	20753.5 ± 1932.8
3.4	182	49	29.1 ± 3.4	6,2 ± 0.6	19402.9 ± 1390.0
3.8	190	57	34.2 ± 9.5	5,4 ± 0.5	19183.7 ± 544.1
2.0	204	71	41.5 ± 2.2	8,0 ± 0.8	18833.5 ± 2361.8
1.9	223	90	17.7 ± 2.0	2,9 ± 0.4	24949.0 ± 2407.3
6.7	233	100	18.0 ± 3.8	2,4 ± 0.3	17368.2 ± 993.8
4.0	238	105	19.9 ± 7.4	1,9 ± 0.3	13902.6 ± 255.7
5.7	243	110	19.6 ± 0.9	2,2 ± 0.3	17917.4 ± 2814.1
3.5	248	115	10.4 ± 3.0	1,7 ± 0.4	12649.1 ± 559.6
3.0	258	125	10.8 ± 2.8	2,0 ± 0.3	4232.5 ± 146.6
4.2	268	135	21.4 ± 5.8	5,1 ± 0.7	2922.4 ± 267.2
4.3	275	142	22.3 ± 5.3	5,6 ± 0.6	4145.9 ± 455.1
5.8	283	150	16.8 ± 3.8	3,2 ± 0.4	4758.6 ± 364.2
4.4	287	154	28.0 ± 1.4	5,8 ± 1.2	4684.6 ± 175.7

3.3	293	160	29.7 ± 9.2	6,6 ± 0.8	5278.4 ± 75.7
5.0	303	170	25.2 ± 6.6	5,0 ± 0.5	10943.1 ± 433.7
4.0	316	183	21.2 ± 4.3	4,7 ± 0.6	7029.2 ± 171.2
4.2	324	191	24.9 ± 0.2	6,0 ± 1.3	3129.9 ± 116.3
7.0	333	200	31.6 ± 8.7	8,4 ± 1.0	3662.6 ± 116.9
5.5	338	205	38.3 ± 8.0	12,6 ± 1.1	5202.2 ± 523.1
5.4	341	208	29.4 ± 4.4	10,4 ± 1.2	4762.0 ± 301.5
8.0	344	211	46.0 ± 2.7	13,4 ± 2.2	4118.1 ± 108.1
8.7	348	215	39.3 ± 8.4	11,9 ± 1.5	4165.7 ± 66.7
11.4	353	220	38.7 ± 11.0	11,5 ± 1.4	5220.1 ± 618.0
7.5	358	225	35.4 ± 7.7	11,1 ± 1.4	4570.2 ± 131.6
