# Supplementary material for: Pharmacokinetics of Snake Antivenom Following Intravenous and Intramuscular Administration in Envenomed Large Animal Model
Source: Pharmaceutics. 2025 Feb 7;17(2):212. doi: 10.3390/pharmaceutics17020212 (PMC11859798; doi:10.3390/pharmaceutics17020212)
Supplement: Supplementary file 1 [file pharmaceutics-17-00212-s001.zip › Supplementary Table S2.pdf]

Table S2. Concentrations of venom, Atx and antivenom measured in the lymph samples of envenomed and *i.v.*-treated sheep.

$L_{i.v.}$ – sheep 1

V / mL	*t* / min	$t_{post\text{-}AV}$ / min	*c*(venom) / ng mL^{-1}	*c*(Atx) / ng mL^{-1}	*c*(antivneom) / µg mL^{-1}
3.0	10		0.0	0.0	
3.7	20		0.0	0.0	
8.4	30		0.0	0.0	
5.0	40		0.0	0.0	
2.9	50		0.0	0.0	
3.4	60		0.0	0.0	
2.9	72		0.0	0.0	
4.0	86		0.0	0.0	
2.3	96		0.0	0.0	
2.5	103		0.0	0.0	
2.5	109		0.0	0.0	
2.9	119		0.0	0.0	
2.0	132	0	0.0	0.0	0.0 ± 0.0
2.3	139	7	0.0	0.0	0.0 ± 0.0
2.5	155	23	0.0	0.0	0.0 ± 0.0
3.5	163	31	0.0	0.0	3.2 ± 0.2
2.5	171	39	0.0	0.0	11.1 ± 0.5
2.4	180	48	0.0	0.0	19.8 ± 1.6
3.5	195	63	0.0	0.0	25.1 ± 1.9
2.4	205	73	0.0	0.0	35.2 ± 0.3
3.0	215	83	0.0	0.0	39.4 ± 1.9
2.8	225	93	0.0	0.0	42.5 ± 3.0
2.2	235	103	0.0	0.0	46.7 ± 1.9
2.5	244	112	0.0	0.0	49.2 ± 1.3
2.5	253	121	0.0	0.0	36.1 ± 1.3
2.9	263	131	0.0	0.0	41.6 ± 2.6
3.5	273	141	0.0	0.0	42.5 ± 5.7
4.5	283	151	0.0	0.0	46.3 ± 3.3
4.4	293	161	0.0	0.0	50.9 ± 3.6
4.0	303	171	0.0	0.0	48.9 ± 2.7
2.5	313	181	0.0	0.0	49.2 ± 1.3
2.9	323	191	0.0	0.0	51.4 ± 0.2
4.0	333	201	0.0	0.0	41.6 ± 0.2
4.0	341	209	0.0	0.0	40.4 ± 0.9
3.0	348	216	0.0	0.0	42.2 ± 0.3
2.4	358	226	0.0	0.0	48.1 ± 3.8
3.5	361	229	0.0	0.0	48.9 ± 2.4
2.5	368	236	0.0	0.0	47.1 ± 1.6
3.0	375	243	0.0	0.0	44.4 ± 2.1
3.9	385	253	0.0	0.0	48.4 ± 2.3
2.4	393	261	0.0	0.0	41.8 ± 3.0

$L_{i.v.}$ – sheep 2

V / mL	*t* / min	$t_{post\text{-}AV}$ / min	*c*(venom) / ng mL^{-1}	*c*(Atx) / ng mL^{-1}	*c*(antivenom) / µg mL^{-1}
13.5	3		0.0 ± 0.0	0.0 ± 0.0	
13.0	6		0.0 ± 0.0	0.0 ± 0.0	
12.5	10		0.0 ± 0.0	0.0 ± 0.0	

13.0	12		12.9 ± 1.2	0.6 ± 0.2	
9.5	15		22.4 ± 2.8	1.2 ± 0.2	
7.0	17		19.0 ± 2.3	1.2 ± 0.1	
9.0	19		15.2 ± 1.7	0.9 ± 0.1	
12.0	24		12.1 ± 1.5	0.6 ± 0.1	
10.0	28		12.3 ± 1.5	0.7 ± 0.1	
11.5	32		21.2 ± 1.9	1.4 ± 0.2	
9.5	37		30.6 ± 2.8	2.3 ± 0.2	
9.0	41		75.0 ± 3.6	4.8 ± 0.5	
6.5	46		91.7 ± 7.3	7.0 ± 0.6	
8.5	49		162.2 ± 20.6	13.4 ± 0.7	
8.0	53		406.1 ± 56.4	33.6 ± 0.6	
8.5	58		507.1 ± 77.3	52.5 ± 0.8	
9.5	64		508.7 ± 85.8	48.0 ± 1.8	
10	68		463.0 ± 41.3	39.5 ± 0.1	
8.0	74		349.9 ± 36.3	30.8 ± 0.3	
14.0	79		246.7 ± 28.1	24.4 ± 0.9	
9.0	89		172.0 ± 31.7	12.2 ± 0.8	
11.0	95		155.1 ± 23.1	10.8 ± 0.7	
8.5	98		149.1 ± 18.8	12.3 ± 1.0	
11.0	102		174.3 ± 22.8	14.2 ± 1.0	
10.0	105		200.8 ± 42.0	14.7 ± 0.9	
10.5	108		201.0 ± 40.5	16.7 ± 0.8	
10.0	112		276.2 ± 39.3	26.8 ± 1.1	
12.0	118		318.3 ± 42.4	27.6 ± 0.6	
13.0	122		209.9 ± 46.9	20.3 ± 1.3	
12.0	127		219.7 ± 52.5	19.4 ± 1.1	
13.5	132	0	331.3 ± 35.4	27.4 ± 0.3	0.0 ± 0.0
14.0	137	5	291.2 ± 30.9	29.5 ± 1.1	0.0 ± 0.0
14.5	144	12	190.5 ± 40.5	16.8 ± 1.4	0.03 ± 0.01
15.0	151	19	150.2 ± 25.9	16.1 ± 1.3	0.1 ± 0.0
12.0	158	26	124.6 ± 16.4	18.0 ± 1.4	0.8 ± 0.1
14.5	165	33	123.1 ± 19.4	16.9 ± 0.8	1.9 ± 0.1
13.0	171	39	120.1 ± 20.1	12.3 ± 0.5	2.0 ± 0.1
14.0	177	45	93.4 ± 14.5	10.7 ± 0.8	4.3 ± 0.3
13.0	183	51	41.4 ± 4.5	7.7 ± 0.8	6.4 ± 0.7
12.5	188	56	33.4 ± 3.9	5.9 ± 0.3	7.8 ± 0.8
12.0	192	60	26.9 ± 3.6	3.5 ± 0.2	8.8 ± 0.7
14.0	198	66	27.0 ± 3.7	3.2 ± 0.3	9.9 ± 1.5
10.0	201	69	28.0 ± 3.5	2.5 ± 0.1	10.0 ± 1.4
11.5	203	71	26.7 ± 3.2	2.9 ± 0.1	9.3 ± 1.4
10.5	208	76	22.3 ± 2.9	2.1 ± 0.1	8.7 ± 1.2
14.0	210	78	26.3 ± 3.4	3.0 ± 0.2	7.3 ± 0.7
13.0	212	80	27.9 ± 3.8	2.7 ± 0.2	9.8 ± 1.5
13.0	216	84	21.8 ± 2.9	1.6 ± 0.0	10.7 ± 1.7
11.0	219	87	15.1 ± 2.6	0.7 ± 0.0	11.2 ± 1.9
12.0	223	91	17.5 ± 2.4	1.0 ± 0.0	11.5 ± 1.8
12.0	228	96	25.1 ± 3.6	1.9 ± 0.0	11.5 ± 1.6
13.5	232	100	26.3 ± 3.4	3.1 ± 0.1	9.1 ± 1.3
13.0	234	102	27.0 ± 2.9	4.4 ± 0.1	7.1 ± 1.0
13.0	238	106	26.2 ± 2.1	3.7 ± 0.1	8.4 ± 1.1
11.5	241	109	36.1 ± 3.8	6.2 ± 0.3	5.1 ± 0.8
13.0	243	111	27.6 ± 3.4	4.0 ± 0.1	7.5 ± 0.8
14.0	245	113	28.1 ± 3.8	4.8 ± 0.1	5.9 ± 0.9

14.0	247	115	24.6 ± 2.8	3.2 ± 0.0	8.0 ± 1.3
13.0	249	117	29.8 ± 3.2	3.7 ± 0.0	7.7 ± 1.1
13.0	251	119	34.3 ± 2.9	5.7 ± 0.2	7.8 ± 1.1
13.5	253	121	31.9 ± 2.4	5.4 ± 0.3	7.1 ± 1.2
13.5	256	124	31.8 ± 2.9	5.3 ± 0.2	7.7 ± 1.1
12.0	258	126	35.7 ± 3.2	6.3 ± 0.2	7.1 ± 1.1
11.0	259	127	34.4 ± 3.4	5.9 ± 0.2	5.8 ± 0.6
13.0	261	129	29.6 ± 2.6	5.2 ± 0.3	7.2 ± 0.9
14.0	262	130	27.7 ± 3.0	4.5 ± 0.2	8.8 ± 1.5
13.5	264	132	32.5 ± 3.3	4.9 ± 0.3	8.4 ± 1.3
15.0	266	134	32.0 ± 3.0	5.6 ± 0.3	7.0 ± 1.1
14.0	268	136	27.5 ± 1.9	5.1 ± 0.4	7.2 ± 0.4
13.0	270	138	28.8 ± 2.9	4.9 ± 0.3	7.9 ± 0.6
12.5	272	140	30.2 ± 3.2	4.3 ± 0.2	8.4 ± 0.7
14.0	274	142	27.5 ± 2.3	3.7 ± 0.1	8.6 ± 0.9
13.5	276	144	25.8 ± 2.5	4.8 ± 0.2	6.3 ± 0.7
14.0	278	146	26.4 ± 2.1	5.0 ± 0.4	6.3 ± 0.3
15.0	280	148	30.3 ± 2.0	6.0 ± 0.3	6.9 ± 0.6
14.0	282	150	27.9 ± 2.2	3.4 ± 0.2	8.3 ± 0.7
14.0	284	152	24.6 ± 1.9	3.8 ± 0.2	7.5 ± 0.8
12.5	286	154	22.4 ± 1.5	2.6 ± 0.2	8.9 ± 1.0
13.0	288	156	25.9 ± 1.7	3.3 ± 0.2	9.5 ± 1.1
14.0	291	159	27.2 ± 2.1	4.1 ± 0.1	7.9 ± 0.6
13.0	294	162	23.5 ± 2.2	3.6 ± 0.1	8.1 ± 0.9
12.5	298	166	22.6 ± 1.2	2.5 ± 0.2	9.8 ± 1.5
14.5	301	169	27.3 ± 2.2	3.7 ± 0.1	7.7 ± 0.7
13.0	304	172	27.7 ± 2.3	4.2 ± 0.0	7.7 ± 0.6
14.0	308	176	24.9 ± 1.8	3.9 ± 0.2	9.1 ± 0.8
14.0	313	181	23.2 ± 1.3	3.9 ± 0.2	8.7 ± 1.1
15.0	318	186	25.0 ± 1.5	3.2 ± 0.1	8.6 ± 1.0
15.0	323	191	25.1 ± 1.8	3.1 ± 0.2	7.7 ± 1.0
13.0	328	196	24.7 ± 1.9	3.3 ± 0.1	7.0 ± 0.7

L$_{i.v.}$ – sheep 3

V / mL	t / min	$t_{\text{post-AV}}$ / min	c(venom) / ng mL^{-1}	c(Atx) / ng mL^{-1}	c(antivenom) / µg mL^{-1}
13.5	3		0.0 ± 0.0	0.0 ± 0.0	
14.5	5		0.0 ± 0.0	0.0 ± 0.0	
10.5	7		0.0 ± 0.0	0.0 ± 0.0	
11.5	9		0.4 ± 0.4	0.0 ± 0.0	
10.0	12		0.5 ± 0.5	0.0 ± 0.0	
9.5	15		1.5 ± 0.8	0.0 ± 0.0	
8.5	17		4.1 ± 2.0	0.0 ± 0.0	
10.0	18		8.6 ± 2.8	0.2 ± 0.1	
9.0	20		9.1 ± 3.3	0.5 ± 0.1	
11.5	22		11.9 ± 2.8	0.5 ± 0.1	
11.5	24		15.8 ± 2.9	0.6 ± 0.2	
10.0	26		26.3 ± 3.9	1.2 ± 0.1	
10.0	28		39.3 ± 5.2	1.9 ± 0.1	
10.5	30		164.2 ± 20.7	7.4 ± 0.8	
12.0	33		318.6 ± 33.0	15.5 ± 1.3	
12.0	36		448.6 ± 50.6	24.2 ± 2.7	
9.5	40		668.8 ± 55.4	38.6 ± 3.1	
9.5	43		862.6 ± 77.7	53.6 ± 4.3	

11.0	47		1176.1 ± 59.1	63.4 ± 4.6	
9.0	51		1712.2 ± 224.3	107.8 ± 4.7	
13.0	54		1574.9 ± 192.5	93.6 ± 3.7	
11.5	57		1325.4 ± 98.3	84.2 ± 5.2	
12.0	60		1371.8 ± 99.7	81.5 ± 5.0	
9.0	64		1363.8 ± 126.3	73.9 ± 4.3	
11.0	67		1156.1 ± 107.6	67.0 ± 1.2	
11.5	69		1078.2 ± 87.6	54.9 ± 2.1	
8.5	71		1087.3 ± 73.6	55.2 ± 3.0	
10.0	73		1099.1 ± 103.9	52.0 ± 3.3	
11.5	76		1011.9 ± 186.2	58.1 ± 5.4	
9.5	79		919.9 ± 98.8	51.3 ± 1.7	
12.0	81		890.0 ± 62.1	49.2 ± 2.9	
10.0	83		873.1 ± 77.6	51.3 ± 4.0	
10.0	85		948.8 ± 136.4	52.3 ± 4.2	
11.5	87		829.3 ± 134.4	46.4 ± 3.4	
7.0	89		811.9 ± 88.3	45.0 ± 4.1	
11.0	91		811.0 ± 64.5	44.3 ± 4.4	
10.0	93		810.5 ± 87.4	46.2 ± 4.2	
10.0	96		803.6 ± 111.2	43.8 ± 4.7	
8.5	98		738.3 ± 79.4	40.0 ± 4.1	
9.0	101		740.8 ± 75.6	41.4 ± 4.9	
8.0	103		774.6 ± 82.5	44.0 ± 3.8	
10.0	106		702.3 ± 79.9	34.1 ± 2.4	
10.0	108		625.3 ± 71.9	30.8 ± 2.9	
13.5	110		606.2 ± 66.8	30.4 ± 3.5	
11.0	112		603.8 ± 54.0	29.3 ± 3.2	
12.0	114		574.1 ± 53.8	28.2 ± 3.1	
10.5	116		578.9 ± 61.9	27.3 ± 2.6	
13.5	118		558.7 ± 65.5	12.3 ± 2.4	
11.0	120		610.9 ± 46.8	29.9 ± 3.0	
11.0	121		614.4 ± 63.8	28.3 ± 1.3	
12.0	123		606.5 ± 71.6	28.0 ± 1.8	
11.5	128		596.2 ± 70.3	28.8 ± 2.7	
10.0	130		636.0 ± 75.3	31.0 ± 3.0	
11.0	132	0	600.7 ± 63.8	26.4 ± 1.7	0.0 ± 0.0
13.0	133	1	549.3 ± 56.1	25.3 ± 1.6	0.0 ± 0.0
13.0	136	4	550.9 ± 64.7	23.7 ± 1.3	0.0 ± 0.0
11.5	138	6	589.4 ± 61.0	26.9 ± 2.4	0.0 ± 0.0
12.0	140	8	552.7 ± 49.1	30.1 ± 1.5	0.0 ± 0.0
15.0	142	10	528.1 ± 55.3	31.4 ± 3.1	0.02 ± 0.01
14.5	145	13	521.6 ± 51.1	30.0 ± 3.1	0.03 ± 0.01
9.0	147	15	662.3 ± 45.4	34.2 ± 1.8	0.03 ± 0.01
10.5	150	18	679.7 ± 61.4	37.6 ± 2.4	0.04 ± 0.02
9.5	152	20	641.1 ± 68.7	40.8 ± 0.8	0.06 ± 0.02
14.0	156	24	616.4 ± 57.8	36.7 ± 2.4	0.1 ± 0.0
10.5	159	27	623.2 ± 54.8	37.6 ± 3.0	0.1 ± 0.0
15.0	161	29	708.2 ± 72.3	40.0 ± 2.2	0.1 ± 0.0
15.0	165	33	592.1 ± 64.8	36.5 ± 1.2	0.7 ± 0.0
14.0	169	37	536.1 ± 46.8	39.3 ± 1.7	1.7 ± 0.1
12.0	172	40	493.5 ± 46.3	40.3 ± 2.3	2.3 ± 0.1
13.5	175	43	487.2 ± 58.0	36.9 ± 2.2	2.5 ± 0.1
14.0	179	47	435.5 ± 85.1	28.8 ± 4.2	3.3 ± 0.2
13.0	183	51	399.7 ± 71.4	36.1 ± 1.9	5.1 ± 0.3

7.0	189	57	317.2 ± 106.7	33.9 ± 1.3	6.2 ± 0.4
15.0	192	60	237.5 ± 65.6	27.6 ± 1.0	8.4 ± 0.6
13.0	194	62	189.8 ± 42.0	22.4 ± 0.1	8.9 ± 0.5
14.0	199	67	250.7 ± 60.6	32.2 ± 0.1	7.7 ± 0.3
14.0	203	71	244.9 ± 73.1	28.5 ± 1.5	9.9 ± 0.5
13.0	205	73	232.4 ± 64.5	26.9 ± 0.6	9.1 ± 0.4
14.0	210	78	197.1 ± 43.4	23.5 ± 0.1	8.7 ± 0.4
14.5	217	85	217.0 ± 46.4	26.3 ± 0.3	7.8 ± 0.2
14.0	219	87	236.3 ± 67.3	15.9 ± 0.5	7.6 ± 0.2
12.0	221	89	229.2 ± 65.7	26.0 ± 0.3	9.0 ± 0.4
10.5	224	92	250.3 ± 30.5	29.0 ± 0.2	9.0 ± 0.5
10.5	227	95	235.2 ± 53.3	29.7 ± 0.4	9.0 ± 0.4
12.5	230	98	267.0 ± 79.4	34.8 ± 0.8	8.5 ± 0.2
11.5	234	102	228.6 ± 60.3	26.5 ± 1.0	9.4 ± 0.3
13.5	238	106	207.2 ± 43.1	25.8 ± 0.5	10.6 ± 0.1
13.0	241	109	187.6 ± 39.5	23.6 ± 0.6	10.1 ± 0.2
11.5	244	112	205.7 ± 56.6	24.5 ± 1.0	10.3 ± 0.2
13.5	248	116	224.5 ± 54.8	28.9 ± 0.7	10.4 ± 0.4
11.0	252	120	230.4 ± 46.8	29.6 ± 0.2	10.8 ± 0.7
10.5	256	124	219.5 ± 47.1	27.0 ± 0.8	12.9 ± 0.2
11.0	259	127	193.4 ± 54.2	23.2 ± 0.3	13.9 ± 0.4
12.0	263	131	188.4 ± 40.8	20.7 ± 0.7	13.6 ± 0.8
11.0	265	133	176.8 ± 34.4	19.8 ± 0.9	12.9 ± 0.7
12.5	269	137	162.8 ± 57.8	22.5 ± 0.8	13.6 ± 0.6
12.0	271	139	160.1 ± 61.3	19.9 ± 0.2	15.3 ± 0.5
12.5	274	142	141.1 ± 50.8	15.7 ± 0.7	16.3 ± 0.4
13.0	277	145	141.4 ± 48.6	16.7 ± 0.8	15.0 ± 0.1
12.5	280	148	139.0 ± 50.8	15.8 ± 1.0	14.5 ± 0.5
14.0	284	152	133.0 ± 48.1	16.6 ± 0.6	15.3 ± 0.7
11.0	286	154	129.7 ± 44.8	15.1 ± 0.2	17.6 ± 0.6
9.5	288	156	141.2 ± 48.3	18.5 ± 0.5	15.5 ± 0.5
11.0	292	160	145.7 ± 52.5	18.6 ± 0.5	16.4 ± 0.1
14.5	296	164	165.0 ± 62.5	23.6 ± 0.6	14.5 ± 0.2
14.0	299	167	206.6 ± 78.5	30.4 ± 1.7	13.8 ± 0.4
13.5	304	172	229.8 ± 84.1	35.9 ± 2.0	13.9 ± 0.3
12.5	309	177	293.4 ± 116.1	48.8 ± 3.2	12.8 ± 0.3
12.0	312	180	270.3 ± 108.7	41.4 ± 2.6	12.2 ± 0.2
12.0	315	183	217.5 ± 83.6	34.8 ± 1.6	13.1 ± 0.3
11.5	319	187	213.2 ± 78.4	34.3 ± 2.3	14.0 ± 0.0
11.5	324	192	225.2 ± 88.9	33.5 ± 2.7	15.1 ± 0.8
15.0	327	195	194.3 ± 78.6	27.6 ± 1.7	15.3 ± 0.6
14.0	329	197	156.0 ± 58.9	17.4 ± 1.2	14.9 ± 0.7
13.0	332	200	158.7 ± 57.3	21.8 ± 1.2	14.6 ± 0.1
13.5	334	202	158.9 ± 61.2	21.0 ± 0.9	14.8 ± 0.3
15.0	338	206	153.6 ± 59.7	20.5 ± 1.3	15.9 ± 0.1
11.0	342	210	145.5 ± 54.1	21.4 ± 1.4	15.0 ± 0.2
14.0	346	214	142.1 ± 50.4	19.3 ± 1.0	14.1 ± 0.3
13.5	348	216	180.1 ± 69.9	25.2 ± 1.2	13.3 ± 0.5
14.0	351	219	155.3 ± 58.6	20.3 ± 2.0	12.2 ± 0.1

$L_{i.v.}$ **– sheep 4**

V / mL	t / min	$t_{post\text{-}AV}$ / min	c(venom) / ng mL^{-1}	c(Atx) / ng mL^{-1}	c(AV) / µg mL^{-1}
9.0	6		0.3 ± 0.3	0.0 ± 0.0	
13.0	11		0.0 ± 0.0	0.0 ± 0.0	
12.4	15		0.0 ± 0.0	0.4 ± 0.4	
11.0	22		0.9 ± 0.9	0.6 ± 0.3	
12.3	27		7.7 ± 3.8	1.3 ± 0.2	
11.7	30		8.6 ± 1.5	3.4 ± 0.3	
9.0	33		7.5 ± 1.7	3.0 ± 1.3	
9.8	37		2.4 ± 1.3	0.6 ± 0.3	
12.0	42		3.4 ± 1.8	0.9 ± 0.4	
8.5	48		3.3 ± 0.8	1.9 ± 0.2	
11.0	54		22.3 ± 6.2	4.5 ± 1.4	
6.3	62		21.7 ± 2.1	7.2 ± 0.6	
10.7	68		44.0 ± 4.9	12.1 ± 0.2	
9.0	75		26.5 ± 3.0	12.1 ± 0.8	
4.0	84		28.9 ± 3.7	11.2 ± 0.6	
2.0	94		46.1 ± 6.0	10.4 ± 0.2	
7.5	117		55.7 ± 4.5	18.6 ± 2.3	
7.5	121		88.7 ± 31.3	25.3 ± 1.2	
8.5	135	0	243.9 ± 31.6	235.7 ± 5.2	0.0 ± 0.0
12.2	139	4	186.2 ± 12.2	122.7 ± 4.7	0.0 ± 0.0
9.5	142	7	123.6 ± 11.4	58.3 ± 0.8	0.0 ± 0.0
9.6	144	9	108.2 ± 33.8	65.0 ± 6.6	0.01 ± 0.0
11.0	147	12	73.2 ± 15.7	76.9 ± 12.6	0.03 ± 0.01
7.5	150	15	63.5 ± 11.8	39.2 ± 5.3	0.05 ± 0.01
10.5	153	18	71.3 ± 7.3	34.6 ± 4.2	0.12 ± 0.01
10.0	158	23	21.1 ± 3.6	26.7 ± 0.5	0.7 ± 0.1
10.0	162	27	5.9 ± 2.4	3.3 ± 0.5	4.2 ± 0.3
10.0	167	32	1.4 ± 1.2	0.0 ± 0.0	7.2 ± 1.3
8.8	172	37	0.6 ± 0.6	0.4 ± 0.2	15.4 ± 1.5
6.5	184	49	0.0 ± 0.0	0.8 ± 0.8	22.5 ± 1.2
7.5	193	58	3.5 ± 1.8	0.5 ± 0.2	16.5 ± 0.8
3.5	203	68	1.2 ± 1.2	0.0 ± 0.0	19.7 ± 0.4
11.0	226	91	1.4 ± 0.8	0.5 ± 0.3	29.4 ± 4.0
9.0	230	95	0.0 ± 0.0	0.0 ± 0.0	31.0 ± 1.0
11.4	234	99	0.0 ± 0.0	0.0 ± 0.0	33.4 ± 2.2
12.0	238	103	0.0 ± 0.0	0.0 ± 0.0	32.5 ± 2.6
11.8	242	107	1.1 ± 1.1	0.0 ± 0.0	34.0 ± 1.0
12.3	246	111	0.4 ± 0.4	0.0 ± 0.0	32.2 ± 1.5
12.5	251	116	0.2 ± 0.2	0.0 ± 0.0	30.7 ± 2.9
10.0	256	121	0.6 ± 0.6	0.0 ± 0.0	30.1 ± 2.4
9.0	261	126	1.6 ± 1.6	0.2 ± 0.2	58.8 ± 6.8
10.0	269	134	2.2 ± 2.2	0.6 ± 0.3	36.5 ± 2.2
10.3	276	141	0.0 ± 0.0	0.0 ± 0.0	65.5 ± 2.7
8.4	293	158	0.9 ± 0.9	0.0 ± 0.0	49.5 ± 0.7

6.5	302	167	1.0 ± 1.0	0.2 ± 0.2	56.4 ± 2.0
12.0	306	171	0.8 ± 0.8	0.2 ± 0.2	42.6 ± 4.9
11.0	313	178	0.6 ± 0.6	0.0 ± 0.0	43.7 ± 3.5
4.5	321	186	0.0 ± 0.0	0.0 ± 0.0	53.2 ± 3.0
