# Supplementary material for: Pharmacokinetics of Snake Antivenom Following Intravenous and Intramuscular Administration in Envenomed Large Animal Model
Source: Pharmaceutics. 2025 Feb 7;17(2):212. doi: 10.3390/pharmaceutics17020212 (PMC11859798; doi:10.3390/pharmaceutics17020212)
Supplement: Supplementary file 1 [file pharmaceutics-17-00212-s001.zip › Supplementary Table S3.pdf]

Table S3. Lymphatic absorption of *i.m.* and *i.v.* antivenoms and its influence on the rate of absorption of *s.c.* injected venom and ammodytoxins (Atxs), as well as on their total amount reaching the lymph during a sampling period ($AUC_{0\text{-}t}$).

	**Route of antivenom administration**			
	***i.m.* (*n* = 4)**		***i.v.* (*n* = 4)**	
	**mean**	**median (IQR)**	**mean**	**median (IQR)**
Antivenom absorption rate [mg h^{-1}]	3.9 ± 1.9	3.0 (1.1 – 7.7)	1.5 ± 0.3	1.5 (0.9 – 2.0)
$AUC_{0\text{-}t}$ (antivenom)* [mg · h]	25.7 ± 14.9	14.9 (5.1 – 57.2)	7.3 ± 1.1	7.2 (5.6 – 9.1)
Venom absorption rate [µg h^{-1}]	17.6 ± 6.9	17.2 (5.0 – 30.6)	41.0 ± 32.1	16.1 (2.2 – 104.7)
$AUC_{0\text{-}t}$ (venom) [µg · h]	465.6 ± 256.6	461.7 (126.0 – 809.2)	853.8 ± 694.6	287.8 (25.5 – 2235.4)
Atx absorption rate [µg h^{-1}]	2.4 ± 1.3	1.9 (0.9 – 5.1)	3.5 ± 1.9	1.7 (1.4 – 7.3)
$AUC_{0\text{-}t}$ (Atx) [µg · h]	63.3 ± 33.1	49.8 (7.7 – 132.3)	62.1 ± 36.5	27.2 (24.0 – 135.2)

*AUC of a mass time graph
