# Supplementary material for: Pharmacokinetics of Snake Antivenom Following Intravenous and Intramuscular Administration in Envenomed Large Animal Model
Source: Pharmaceutics. 2025 Feb 7;17(2):212. doi: 10.3390/pharmaceutics17020212 (PMC11859798; doi:10.3390/pharmaceutics17020212)
Supplement: Supplementary file 1 [file pharmaceutics-17-00212-s001.zip › Supplementary Table S4.pdf]

Table S4. Concentrations of venom, Atx and antivenom measured in the serum samples of envenomed and *i.m.*-treated sheep.

$S_{i.m.}$ – sheep 1

t / h	$t_{post-AV}$ / h	*c*(venom) / ng mL^{-1}	*c*(Atx) / ng mL^{-1}	*c*(antivenom) / µg mL^{-1}
0.04		0.0 ± 0.0	0.0 ± 0.0	
0.08		16.0 ± 2.8	0.1 ± 0.1	
0.13		21.3 ± 3.2	0.5 ± 0.1	
0.17		22.4 ± 5.4	0.7 ± 0.1	
0.25		20.5 ± 2.9	0.6 ± 0.1	
0.33		33.2 ± 2.4	0.9 ± 0.1	
0.50		45.9 ± 5.2	1.7 ± 0.2	
0.67		42.3 ± 6.4	1.6 ± 0.1	
0.83		35.3 ± 4.0	1.0 ± 0.2	
1.00		36.0 ± 2.2	0.7 ± 0.2	
1.25		33.6 ± 3.5	0.7 ± 0.1	
1.50		30.5 ± 4.4	0.7 ± 0.1	
1.75		27.5 ± 2.4	0.6 ± 0.1	
2.00		37.3 ± 4.5	0.7 ± 0.1	
2.25	0.05	38.1 ± 2.9	0.9 ± 0.1	0.00 ± 0.0
2.33	0.13	29.5 ± 4.6	0.6 ± 0.3	0.00 ± 0.0
2.42	0.22	23.6 ± 2.8	0.4 ± 0.2	0.00 ± 0.0
2.50	0.30	20.7 ± 2.8	0.4 ± 0.2	0.00 ± 0.0
2.67	0.47	24.7 ± 3.6	0.7 ± 0.2	0.01 ± 0.0
2.83	0.63	17.8 ± 2.9	0.5 ± 0.2	0.05 ± 0.0
3.00	0.80	13.0 ± 2.2	0.3 ± 0.2	0.1 ± 0.0
3.17	0.97	10.8 ± 1.9	0.3 ± 0.2	0.2 ± 0.0
3.42	1.22	15.4 ± 3.0	0.4 ± 0.3	0.2 ± 0.0
3.67	1.47	10.6 ± 3.0	0.3 ± 0.2	0.7 ± 0.0
3.92	1.72	6.8 ± 1.1	0.0 ± 0.0	1.7 ± 0.1
4.17	1.97	0.0 ± 0.0	0.0 ± 0.0	2.5 ± 0.1
4.67	2.47	0.0 ± 0.0	0.0 ± 0.0	6.1 ± 0.3
5.17	2.97	0.0 ± 0.0	0.0 ± 0.0	9.3 ± 0.6
5.67	3.47	0.0 ± 0.0	0.0 ± 0.0	13.5 ± 0.3
6.17	3.97	0.0 ± 0.0	0.0 ± 0.0	17.7 ± 0.7
6.67	4.47	0.0 ± 0.0	0.0 ± 0.0	21.4 ± 1.1
7.17	4.97	0.0 ± 0.0	0.0 ± 0.0	28.1 ± 1.4
7.67	5.47	0.0 ± 0.0	0.0 ± 0.0	39.1 ± 2.0
8.17	5.97	0.0 ± 0.0	0.0 ± 0.0	31.8 ± 1.0
12	9.8	0.0 ± 0.0	0.0 ± 0.0	68.1 ± 2.6
18	15.8	0.0 ± 0.0	0.0 ± 0.0	86.4 ± 3.6
24	21.8	12.7 ± 2.4	0.0 ± 0.0	78.4 ± 3.3
48	45.8	22.2 ± 6.6	0.0 ± 0.0	61.7 ± 1.8
72	69.8	17.7 ± 4.1	0.0 ± 0.0	50.6 ± 2.1
96	93.8	3.8 ± 1.3	0.0 ± 0.0	43.7 ± 1.9
120	117.8	0.0 ± 0.0	0.0 ± 0.0	37.6 ± 0.8

144	141.8	0.0 ± 0.0	0.0 ± 0.0	22.4 ± 0.8
168	165.8	0.0 ± 0.0	0.0 ± 0.0	12.8 ± 0.6
192	189.8	0.0 ± 0.0	0.0 ± 0.0	5.3 ± 0.4
216	213.8	0.0 ± 0.0	0.0 ± 0.0	3.1 ± 0.2
240	237.8	0.0 ± 0.0	0.0 ± 0.0	2.4 ± 0.1
264	261.8	0.0 ± 0.0	0.0 ± 0.0	1.7 ± 0.1
288	285.8	0.0 ± 0.0	0.0 ± 0.0	1.2 ± 0.1
312	309.8	0.0 ± 0.0	0.0 ± 0.0	0.6 ± 0.1

$S_{i.m.}$ – sheep 2

t / h	$t_{post\text{-}AV}$ / h	c(venom) / ng mL^{-1}	c(Atx) / ng mL^{-1}	c(antivenom) / μg mL^{-1}
0.04		11.9 ± 1.0	0.8 ± 0.5	
0.08		16.4 ± 1.1	1.0 ± 0.4	
0.13		21.6 ± 1.2	1.4 ± 0.4	
0.17		24.9 ± 2.7	2.0 ± 0.5	
0.25		27.8 ± 1.6	2.2 ± 0.6	
0.33		29.0 ± 2.3	2.3 ± 0.6	
0.50		47.1 ± 4.4	4.0 ± 0.8	
0.67		58.9 ± 3.0	5.0 ± 0.9	
0.83		64.5 ± 4.2	5.2 ± 0.9	
1.00		71.6 ± 6.3	5.6 ± 1.2	
1.25		73.3 ± 5.4	5.4 ± 0.9	
1.50		70.5 ± 3.2	5.0 ± 0.8	
1.75		72.4 ± 5.1	4.8 ± 0.9	
2.00		81.6 ± 6.2	5.2 ± 1.1	
2.25	0.05	74.7 ± 5.0	4.6 ± 0.8	0.0 ± 0.0
2.33	0.1	70.7 ± 4.7	4.6 ± 0.9	0.0 ± 0.0
2.42	0.2	66.7 ± 2.7	4.6 ± 0.8	0.0 ± 0.0
2.50	0.3	56.8 ± 5.3	4.4 ± 0.9	0.0 ± 0.0
2.67	0.5	41.7 ± 2.3	3.5 ± 0.5	0.3 ± 0.0
2.83	0.6	31.3 ± 2.4	2.3 ± 0.6	0.5 ± 0.1
3.00	0.8	28.7 ± 2.3	2.3 ± 0.5	1.2 ± 0.1
3.17	1.0	21.0 ± 1.2	1.6 ± 0.5	2.2 ± 0.0
3.42	1.2	13.8 ± 1.2	0.7 ± 0.4	3.3 ± 0.1
3.67	1.5	10.5 ± 1.3	0.5 ± 0.3	4.9 ± 0.0
3.92	1.7	8.6 ± 0.7	0.4 ± 0.2	7.1 ± 0.3
4.17	2.0	8.0 ± 1.1	0.3 ± 0.2	8.2 ± 0.2
4.67	2.5	6.5 ± 0.5	0.2 ± 0.1	11.2 ± 0.4
5.17	3.0	7.6 ± 1.1	0.3 ± 0.0	13.6 ± 0.2
5.67	3.5	10.0 ± 1.0	0.3 ± 0.1	15.9 ± 0.6
6.17	4.0	11.6 ± 1.4	0.5 ± 0.2	18.6 ± 0.3
6.67	4.5	11.9 ± 1.4	0.5 ± 0.1	19.5 ± 0.6
7.17	5.0	13.3 ± 1.3	0.5 ± 0.1	20.3 ± 0.5
7.67	5.5	16.8 ± 1.9	0.9 ± 0.2	22.6 ± 1.1
8.17	6.0	17.2 ± 2.0	0.9 ± 0.4	21.5 ± 0.1
12	9.8	27.2 ± 2.1	1.9 ± 0.4	22.1 ± 0.2

18	15.8	28.8 ± 2.7	1.8 ± 0.3	22.6 ± 0.6
24	21.8	26.0 ± 3.1	1.0 ± 0.3	27.1 ± 0.3
48	45.8	25.8 ± 2.9	0.8 ± 0.0	38.8 ± 0.4
72	69.8	13.3 ± 1.8	0.2 ± 0.0	38.6 ± 1.0
96	93.8	0.0 ± 0.0	0.1 ± 0.0	34.8 ± 1.3
120	117.8	0.0 ± 0.0	0.2 ± 0.0	32.1 ± 1.1
144	141.8	0.0 ± 0.0	0.2 ± 0.1	23.9 ± 0.3
168	165.8	0.0 ± 0.0	0.3 ± 0.1	18.3 ± 0.8
192	189.8	0.0 ± 0.0	0.3 ± 0.1	9.5 ± 0.1
216	213.8	0.0 ± 0.0	0.4 ± 0.1	4.7 ± 0.3
240	237.8	0.0 ± 0.0	0.4 ± 0.0	2.2 ± 0.2
264	261.8	0.0 ± 0.0	0.3 ± 0.0	0.4 ± 0.0
288	285.8	0.0 ± 0.0	0.3 ± 0.0	0.1 ± 0.0
312	309.8	0.0 ± 0.0	0.4 ± 0.0	0.1 ± 0.0

$S_{i.m.}$ – sheep 3

t / h	$t_{post\text{-}AV}$ / h	c(venom) / ng mL^{-1}	c(Atx) / ng mL^{-1}	c(antivenom) / µg mL^{-1}
0.04		8.6 ± 0.7	0.0 ± 0.0	
0.08		10.6 ± 1.4	0.0 ± 0.0	
0.13		12.0 ± 1.5	1.9 ± 0.3	
0.17		12.6 ± 1.4	2.0 ± 0.2	
0.25		15.1 ± 1.1	2.3 ± 0.2	
0.33		17.2 ± 2.0	2.6 ± 0.3	
0.50		22.0 ± 3.1	3.5 ± 0.5	
0.67		31.1 ± 3.4	4.9 ± 0.5	
0.83		34.6 ± 2.6	5.1 ± 0.7	
1.00		39.0 ± 4.5	5.4 ± 0.6	
1.25		42.5 ± 3.9	6.0 ± 0.6	
1.50		44.6 ± 1.4	6.0 ± 0.5	
1.75		47.7 ± 4.3	5.5 ± 0.6	
2.00		47.8 ± 4.4	5.8 ± 0.6	
2.25	0.05	46.9 ± 3.2	6.9 ± 0.7	0.0 ± 0.0
2.33	0.1	48.5 ± 5.2	6.6 ± 0.5	0.0 ± 0.0
2.42	0.2	35.0 ± 0.9	5.5 ± 0.7	0.0 ± 0.0
2.50	0.3	34.3 ± 2.9	5.2 ± 0.8	0.0 ± 0.0
2.67	0.5	34.0 ± 2.8	5.6 ± 0.8	0.0 ± 0.0
2.83	0.6	28.6 ± 2.1	5.0 ± 0.7	0.2 ± 0.0
3.00	0.8	25.2 ± 0.6	4.6 ± 0.6	0.6 ± 0.0
3.17	1.0	25.4 ± 3.3	4.5 ± 0.6	0.9 ± 0.1
3.42	1.2	22.6 ± 2.8	4.5 ± 0.7	1.4 ± 0.1
3.67	1.5	18.2 ± 1.7	3.6 ± 0.5	2.1 ± 0.0
3.92	1.7	16.2 ± 2.0	3.0 ± 0.4	3.2 ± 0.3
4.17	2.0	14.8 ± 2.0	2.5 ± 0.3	4.3 ± 0.4
4.67	2.5	10.3 ± 0.8	1.8 ± 0.2	5.9 ± 0.6
5.17	3.0	9.5 ± 1.6	0.0 ± 0.0	8.1 ± 0.8
5.67	3.5	9.0 ± 1.8	0.0 ± 0.0	10.9 ± 0.9

6.17	4.0	9.3 ± 1.3	0.0 ± 0.0	12.9 ± 0.4
6.67	4.5	7.7 ± 0.6	0.0 ± 0.0	13.3 ± 0.5
7.17	5.0	8.2 ± 1.4	0.0 ± 0.0	15.5 ± 0.4
7.67	5.5	8.7 ± 1.6	0.0 ± 0.0	18.7 ± 0.5
8.17	6.0	8.1 ± 1.4	0.0 ± 0.0	20.6 ± 0.4
12	9.8	7.9 ± 0.9	0.0 ± 0.0	23.6 ± 0.6
18	15.8	9.4 ± 1.1	0.0 ± 0.0	27.6 ± 0.6
24	21.8	10.4 ± 1.2	0.0 ± 0.0	33.8 ± 1.1
48	45.8	6.6 ± 0.8	0.0 ± 0.0	37.0 ± 3.4
72	69.8	5.8 ± 1.0	0.0 ± 0.0	26.2 ± 1.9
96	93.8	0.0 ± 0.0	0.0 ± 0.0	21.3 ± 1.9
120	117.8	0.0 ± 0.0	0.0 ± 0.0	16.7 ± 0.4
144	141.8	0.0 ± 0.0	0.0 ± 0.0	5.7 ± 0.4
168	165.8	5.8 ± 2.0	0.0 ± 0.0	0.3 ± 0.0
192	189.8	9.2 ± 2.7	1.4 ± 0.3	0.1 ± 0.0
216	213.8	7.4 ± 2.3	1.8 ± 0.0	0.0 ± 0.0
240	237.8	6.6 ± 2.5	0.0 ± 0.0	0.0 ± 0.0
264	261.8	7.4 ± 2.4	0.0 ± 0.0	0.0 ± 0.0
288	285.8	6.8 ± 2.0	0.0 ± 0.0	0.0 ± 0.0
312	309.8	0.0 ± 0.0	0.0 ± 0.0	0.0 ± 0.0

S*i.m.* – sheep 4

t / h	$t_{\text{post-AV}}$ / h	*c*(venom) / ng mL^{-1}	*c*(Atx) / ng mL^{-1}	*c*(antivenom) / μg mL^{-1}
0.04		2.6 ± 1.1	0.0 ± 0.0	
0.08		4.8 ± 1.2	3.3 ± 1.1	
0.13		3.6 ± 1.2	2.2 ± 0.2	
0.17		3.5 ± 1.2	2.4 ± 0.6	
0.25		4.9 ± 1.0	2.0 ± 0.6	
0.33		11.5 ± 1.5	5.4 ± 0.3	
0.50		11.4 ± 1.7	4.3 ± 0.0	
0.67		18.6 ± 1.6	6.1 ± 0.1	
0.83		35.7 ± 2.1	11.8 ± 0.1	
1.00		55.4 ± 2.8	13.1 ± 0.9	
1.25		54.2 ± 2.8	14.6 ± 2.7	
1.50		56.2 ± 2.1	16.8 ± 1.2	
1.75		54.3 ± 2.7	13.3 ± 0.9	
2.00		62.2 ± 0.4	12.6 ± 0.1	
2.25	0.05	64.5 ± 2.9	16.4 ± 0.9	0.0 ± 0.0
2.33	0.1	58.2 ± 1.9	21.9 ± 0.8	0.0 ± 0.0
2.42	0.2	48.3 ± 1.5	18.8 ± 0.6	0.0 ± 0.0
2.50	0.3	50.9 ± 0.1	16.0 ± 0.1	0.1 ± 0.0
2.67	0.5	46.6 ± 5.1	20.3 ± 2.3	0.3 ± 0.1
2.83	0.6	32.6 ± 1.9	15.0 ± 1.4	1.3 ± 0.1
3.00	0.8	19.9 ± 2.0	5.8 ± 0.1	3.0 ± 0.1
3.17	1.0	7.8 ± 1.2	3.1 ± 1.0	7.8 ± 0.5
3.42	1.2	4.6 ± 1.3	3.6 ± 1.0	13.0 ± 0.4

3.67	1.5	8.1 ± 1.2	2.3 ± 0.4	14.4 ± 0.2
3.92	1.7	13.4 ± 1.4	4.5 ± 0.1	16.1 ± 0.5
4.17	2.0	20.5 ± 1.7	6.6 ± 0.7	17.9 ± 0.2
4.67	2.5	16.4 ± 2.2	6.4 ± 0.3	23.5 ± 0.6
5.17	3.0	13.9 ± 1.4	4.6 ± 0.0	26.4 ± 1.1
5.67	3.5	16.1 ± 1.8	5.6 ± 0.0	28.1 ± 0.6
6.17	4.0	16.3 ± 1.6	5.1 ± 0.8	34.3 ± 1.3
6.67	4.5	11.7 ± 1.8	3.4 ± 0.0	35.3 ± 1.2
7.17	5.0	10.9 ± 1.4	3.2 ± 0.1	36.3 ± 2.7
7.67	5.5	18.4 ± 1.8	5.9 ± 0.3	36.5 ± 2.1
8.17	6.0	22.4 ± 2.7	8.2 ± 0.8	35.3 ± 2.0
12	9.8	26.9 ± 2.8	7.9 ± 0.6	42.3 ± 1.1
18	15.8	16.2 ± 1.8	3.7 ± 0.1	46.3 ± 2.2
24	21.8	19.2 ± 2.0	4.5 ± 0.1	47.2 ± 1.7
48	45.8	11.1 ± 1.5	2.4 ± 0.1	45.2 ± 1.9
72	69.8	2.0 ± 1.5	0.0 ± 0.0	38.5 ± 0.7
96	93.8	0.0 ± 0.0	0.0 ± 0.0	34.4 ± 2.5
120	117.8	0.0 ± 0.0	0.0 ± 0.0	28.4 ± 1.7
144	141.8	0.0 ± 0.0	2.2 ± 0.0	24.5 ± 2.1
168	165.8	0.0 ± 0.0	1.5 ± 0.4	15.7 ± 0.7
192	189.8	0.0 ± 0.0	0.0 ± 0.0	9.5 ± 0.3
216	213.8	0.0 ± 0.0	0.0 ± 0.0	6.5 ± 0.4
240	237.8	0.0 ± 0.0	0.0 ± 0.0	4.5 ± 0.1
312	309.8	0.0 ± 0.0	0.0 ± 0.0	2.1 ± 0.2
