# Supplementary material for: Pharmacokinetics of Snake Antivenom Following Intravenous and Intramuscular Administration in Envenomed Large Animal Model
Source: Pharmaceutics. 2025 Feb 7;17(2):212. doi: 10.3390/pharmaceutics17020212 (PMC11859798; doi:10.3390/pharmaceutics17020212)
Supplement: Supplementary file 1 [file pharmaceutics-17-00212-s001.zip › Supplementary Table S5.pdf]

Table S5. Concentrations of venom, Atx and antivenom measured in the serum samples of envenomed and *i.v.*-treated sheep.

$S_{i.v.}$ – sheep 1

t / h	$t_{post\text{-}AV}$ / h	*c*(venom) / ng mL^{-1}	*c*(Atx) / ng mL^{-1}	*c*(antivenom) / µg mL^{-1}
0.04		16.2 ± 3.3	0.6 ± 0.1	
0.08		24.1 ± 4.7	0.9 ± 0.1	
0.20		21.2 ± 3.7	1.2 ± 0.1	
0.25		21.6 ± 2.2	1.2 ± 0.1	
0.28		24.6 ± 2.6	1.4 ± 0.0	
0.33		38.1 ± 3.4	1.9 ± 0.1	
0.42		38.7 ± 3.2	2.2 ± 0.1	
0.50		37.7 ± 5.0	2.2 ± 0.0	
0.67		47.9 ± 2.1	2.8 ± 0.1	
0.83		52.7 ± 1.2	2.7 ± 0.2	
1.00		45.9 ± 5.9	2.4 ± 0.1	
1.25		39.6 ± 3.9	2.3 ± 0.2	
1.50		37.2 ± 2.2	1.9 ± 0.2	
1.75		47.7 ± 4.7	2.3 ± 0.3	
2.00		45.6 ± 5.9	2.0 ± 0.1	
2.25	0.05	38.9 ± 4.7	1.9 ± 0.1	0.0 ± 0.0
2.33	0.13	0.0 ± 0.0	0.0 ± 0.0	4.3 ± 0.2
2.42	0.22	0.0 ± 0.0	0.0 ± 0.0	51.4 ± 1.4
2.50	0.30	0.0 ± 0.0	0.0 ± 0.0	87.1 ± 3.3
2.67	0.47	0.0 ± 0.0	0.0 ± 0.0	246.6 ± 11.7
2.83	0.63	0.0 ± 0.0	0.0 ± 0.0	301.4 ± 13.8
3.00	0.80	0.0 ± 0.0	0.0 ± 0.0	310.7 ± 10.8
3.17	0.97	0.0 ± 0.0	0.0 ± 0.0	322.7 ± 17.3
3.42	1.22	0.0 ± 0.0	0.0 ± 0.0	310.0 ± 8.8
3.67	1.47	0.0 ± 0.0	0.0 ± 0.0	311.0 ± 12.4
3.92	1.72	0.0 ± 0.0	0.0 ± 0.0	320.4 ± 18.3
4.17	1.97	0.0 ± 0.0	0.0 ± 0.0	278.9 ± 7.5
4.67	2.47	0.0 ± 0.0	0.0 ± 0.0	282.6 ± 10.2
5.17	2.97	0.0 ± 0.0	0.0 ± 0.0	260.4 ± 1.2
5.67	3.47	0.0 ± 0.0	0.0 ± 0.0	276.5 ± 8.6
6.17	3.97	0.0 ± 0.0	0.0 ± 0.0	275.8 ± 15.1
6.67	4.47	0.0 ± 0.0	0.0 ± 0.0	257.0 ± 18.4
7.17	4.97	0.0 ± 0.0	0.0 ± 0.0	247.4 ± 8.9
7.67	5.47	0.0 ± 0.0	0.0 ± 0.0	260.2 ± 15.8
8.17	5.97	0.0 ± 0.0	0.0 ± 0.0	214.6 ± 12.1
12	9.8	0.0 ± 0.0	0.0 ± 0.0	163.4 ± 8.2
18	15.8	17.2 ± 1.5	0.0 ± 0.0	112.7 ± 12.3
24	21.8	24.4 ± 2.9	2.2 ± 0.1	78.7 ± 7.1
48	45.8	31.4 ± 4.0	1.1 ± 0.1	56.1 ± 2.9
72	69.8	33.7 ± 1.1	1.0 ± 0.1	49.3 ± 2.1
96	93.8	15.9 ± 2.8	0.0 ± 0.0	36.9 ± 1.2

120	117.8	16.5 ± 2.8	0.0 ± 0.0	33.9 ± 1.7
144	141.8	17.7 ± 4.4	0.0 ± 0.0	20.5 ± 1.1
168	165.8	17.6 ± 5.6	1.1 ± 0.2	7.5 ± 0.4
192	189.8	16.9 ± 5.5	1.0 ± 0.2	3.9 ± 0.3
216	213.8	0.0 ± 0.0	0.0 ± 0.0	2.4 ± 0.2
240	237.8	0.0 ± 0.0	0.0 ± 0.0	1.5 ± 0.2
264	261.8	0.0 ± 0.0	1.3 ± 0.2	1.0 ± 0.1
288	285.8	0.0 ± 0.0	1.2 ± 0.2	0.7 ± 0.1
312	309.8	0.0 ± 0.0	0.0 ± 0.0	0.7 ± 0.1

$S_{i.v.}$ – sheep 2

t / h	$t_{post\text{-}AV}$ / h	c(venom) / ng mL^{-1}	c(Atx) / ng mL^{-1}	c(antivenom) / µg mL^{-1}
0.13		19.2 ± 1.3	2.2 ± 0.6	
0.30		35.3 ± 2.6	3.9 ± 1.2	
0.50		46.0 ± 3.9	5.8 ± 1.7	
0.67		53.4 ± 5.3	5.8 ± 1.7	
0.83		66.1 ± 6.0	6.2 ± 1.9	
1.0		71.0 ± 4.7	7.0 ± 2.2	
1.3		70.9 ± 7.0	7.9 ± 2.3	
1.5		65.9 ± 7.3	6.4 ± 2.0	
1.8		71.6 ± 8.3	6.5 ± 2.1	
2.0		71.1 ± 4.3	5.7 ± 1.7	
2.8	0.17	0.0 ± 0.0	0.0 ± 0.0	177.7 ± 7.0
2.8	0.3	0.0 ± 0.0	0.0 ± 0.0	178.3 ± 3.3
2.9	0.3	0.0 ± 0.0	0.0 ± 0.0	194.5 ± 4.1
3.0	0.4	0.0 ± 0.0	0.0 ± 0.0	160.3 ± 10.7
3.1	0.5	0.0 ± 0.0	0.0 ± 0.0	155.0 ± 8.0
3.3	0.7	0.0 ± 0.0	0.0 ± 0.0	157.3 ± 10.9
3.4	0.8	0.0 ± 0.0	0.0 ± 0.0	155.6 ± 10.4
3.6	1.0	0.0 ± 0.0	0.0 ± 0.0	168.3 ± 6.7
3.8	1.2	0.0 ± 0.0	0.0 ± 0.0	150.3 ± 4.3
4.0	1.4	0.0 ± 0.0	0.0 ± 0.0	149.7 ± 2.5
4.3	1.7	0.0 ± 0.0	0.0 ± 0.0	158.2 ± 3.6
4.5	1.9	0.0 ± 0.0	0.0 ± 0.0	130.7 ± 8.3
4.8	2.2	0.0 ± 0.0	0.0 ± 0.0	121.3 ± 6.1
5.3	2.7	0.0 ± 0.0	0.0 ± 0.0	119.0 ± 5.2
5.8	3.2	0.0 ± 0.0	0.0 ± 0.0	118.0 ± 5.0
6.3	3.7	0.0 ± 0.0	0.0 ± 0.0	121.6 ± 5.5
6.8	4.2	0.0 ± 0.0	0.0 ± 0.0	104.4 ± 3.7
7.3	4.7	0.0 ± 0.0	0.0 ± 0.0	100.1 ± 3.7
7.8	5.2	3.1 ± 1.7	0.0 ± 0.0	101.4 ± 4.2
8.3	5.7	8.8 ± 1.5	0.0 ± 0.0	79.8 ± 5.9
12	9.4	24.9 ± 2.7	5.4 ± 1.7	57.4 ± 2.6
18	15.4	33.5 ± 4.0	5.7 ± 2.0	39.0 ± 1.7
24	21.4	38.1 ± 4.6	5.3 ± 1.9	31.1 ± 1.6
48	45.4	40.6 ± 4.3	2.9 ± 0.9	25.0 ± 1.9

72	69.4	31.6 ± 2.6	1.5 ± 0.6	20.6 ± 1.0
96	93.4	17.0 ± 2.1	0.0 ± 0.0	17.2 ± 1.3
120	117.4	9.8 ± 1.1	0.0 ± 0.0	12.9 ± 0.3
144	141.4	8.3 ± 0.9	0.0 ± 0.0	10.7 ± 0.9
168	165.4	9.0 ± 1.4	0.0 ± 0.0	6.4 ± 0.3
192	189.4	3.6 ± 2.1	0.0 ± 0.0	3.3 ± 0.1
216	213.4	7.1 ± 1.6	0.7 ± 0.3	1.9 ± 0.1
240	237.4	5.5 ± 1.0	0.0 ± 0.0	1.3 ± 0.1
264	261.4	0.0 ± 0.0	0.0 ± 0.0	0.8 ± 0.0
288	285.4	0.0 ± 0.0	0.0 ± 0.0	0.5 ± 0.0
312	309.4	0.0 ± 0.0	0.0 ± 0.0	0.3 ± 0.0

S*i.v.* – sheep 3

t / h	$t_{post\text{-}AV}$ / h	*c*(venom) / ng mL^{-1}	*c*(Atx) / ng mL^{-1}	*c*(antivenom) / μg mL^{-1}
0.0		0.0 ± 0.0	0.0 ± 0.0	
0.04		0.0 ± 0.0	0.0 ± 0.0	
0.08		0.0 ± 0.0	2.4 ± 0.3	
0.13		3.1 ± 1.6	3.4 ± 0.5	
0.17		0.0 ± 0.0	2.1 ± 0.1	
0.25		0.0 ± 0.0	2.9 ± 0.3	
0.33		3.5 ± 3.5	3.9 ± 0.2	
0.50		4.7 ± 4.7	4.3 ± 0.6	
0.67		0.0 ± 0.0	2.6 ± 0.2	
0.83		2.7 ± 2.7	3.9 ± 0.4	
1.0		3.2 ± 3.2	5.6 ± 0.4	
1.3		5.1 ± 5.1	6.2 ± 0.6	
1.5		3.0 ± 3.0	3.8 ± 0.2	
1.8		3.5 ± 3.5	4.3 ± 0.5	
2.0		4.4 ± 4.4	5.6 ± 0.7	
2.3	0.1	0.0 ± 0.0	0.0 ± 0.0	47.6 ± 2.9
2.3	0.1	0.0 ± 0.0	0.0 ± 0.0	85.4 ± 3.5
2.4	0.2	0.0 ± 0.0	0.0 ± 0.0	95.6 ± 4.7
2.5	0.3	0.0 ± 0.0	3.5 ± 0.8	105.3 ± 5.9
2.7	0.5	0.0 ± 0.0	0.0 ± 0.0	150.3 ± 7.7
2.8	0.6	0.0 ± 0.0	0.0 ± 0.0	156.8 ± 4.2
3.0	0.8	0.0 ± 0.0	0.0 ± 0.0	162.1 ± 7.4
3.2	1.0	0.0 ± 0.0	2.8 ± 0.7	128.2 ± 6.4
3.4	1.2	0.0 ± 0.0	0.0 ± 0.0	120.1 ± 7.0
3.7	1.5	0.0 ± 0.0	0.0 ± 0.0	119.4 ± 4.0
3.9	1.7	0.0 ± 0.0	0.0 ± 0.0	119.4 ± 10.5
4.2	2.0	0.0 ± 0.0	2.1 ± 0.2	112.6 ± 4.8
4.7	2.5	0.0 ± 0.0	0.0 ± 0.0	111.5 ± 5.1
5.2	3.0	0.0 ± 0.0	0.0 ± 0.0	113.0 ± 4.8
5.7	3.5	0.0 ± 0.0	0.0 ± 0.0	108.5 ± 10.6

6.2	4.0	0.0 ± 0.0	0.0 ± 0.0	98.9 ± 4.1
6.7	4.5	0.0 ± 0.0	0.0 ± 0.0	89.5 ± 4.3
7.2	5.0	0.0 ± 0.0	0.0 ± 0.0	91.3 ± 7.8
7.7	5.5	0.0 ± 0.0	0.0 ± 0.0	82.0 ± 6.5
8.2	6.0	0.0 ± 0.0	0.0 ± 0.0	77.8 ± 2.6
12	9.8	0.0 ± 0.0	0.0 ± 0.0	77.5 ± 5.6
18	15.8	12.6 ± 0.4	2.8 ± 0.3	47.8 ± 2.9
24	21.8	26.1 ± 1.0	10.7 ± 1.3	37.9 ± 1.5
48	45.8	32.5 ± 0.5	10.0 ± 1.5	26.4 ± 1.2
72	69.8	25.9 ± 1.6	4.9 ± 0.4	23.4 ± 1.6
96	93.8	5.5 ± 0.1	0.0 ± 0.0	16.2 ± 1.7
120	117.8	0.0 ± 0.0	0.0 ± 0.0	16.5 ± 1.2
144	141.8	0.0 ± 0.0	1.4 ± 0.3	10.8 ± 2.0
168	165.8	0.0 ± 0.0	2.3 ± 0.1	4.5 ± 0.2
192	189.8	0.0 ± 0.0	0.0 ± 0.0	2.9 ± 0.2
216	213.8	0.0 ± 0.0	0.0 ± 0.0	2.4 ± 0.1
288	285.8	0.0 ± 0.0	1.8 ± 0.1	0.6 ± 0.0
312	309.8	0.0 ± 0.0	2.9 ± 0.2	0.5 ± 0.0

$S_{i.v.}$ – sheep 4

***t* / h**	**$t_{post\text{-}AV}$ / h**	***c*(venom) / ng mL^{-1}**	***c*(Atx) / ng mL^{-1}**	***c*(antivenom) / μg mL^{-1}**
0.04		19.9 ± 1.5	5.4 ± 0.4	
0.08		26.4 ± 2.5	6.9 ± 0.6	
0.13		34.5 ± 1.7	9.2 ± 0.8	
0.17		28.3 ± 2.3	7.9 ± 1.1	
0.25		27.2 ± 1.9	7.3 ± 0.7	
0.3		42.7 ± 1.9	8.4 ± 0.8	
0.5		53.8 ± 4.7	11.6 ± 1.4	
0.7		67.0 ± 2.4	11.0 ± 2.1	
0.8		52.4 ± 3.2	10.6 ± 1.5	
1.0		54.0 ± 3.3	11.6 ± 1.8	
1.3		66.8 ± 4.6	12.9 ± 1.6	
1.5		68.1 ± 2.4	12.2 ± 2.2	
1.8		58.5 ± 3.9	11.5 ± 1.6	
2.0		62.9 ± 3.9	12.4 ± 1.8	
2.5	0.05	0.0 ± 0.0	2.0 ± 0.8	76.8 ± 6.3
2.6	0.2	0.0 ± 0.0	2.6 ± 0.5	78.2 ± 2.2
2.7	0.3	0.0 ± 0.0	2.0 ± 0.9	180.0 ± 14.0
2.9	0.5	0.0 ± 0.0	3.4 ± 1.3	142.9 ± 12.2
3.0	0.6	0.0 ± 0.0	1.7 ± 0.6	130.7 ± 15.4
3.2	0.8	0.0 ± 0.0	3.0 ± 1.1	140.9 ± 3.5
3.3	0.9	0.0 ± 0.0	0.0 ± 0.0	131.2 ± 3.9
3.4	1.0	0.0 ± 0.0	0.8 ± 0.8	124.9 ± 8.4
3.7	1.3	0.0 ± 0.0	1.7 ± 1.7	113.5 ± 7.3
3.9	1.5	0.0 ± 0.0	0.0 ± 0.0	115.5 ± 12.5
4.2	1.8	0.0 ± 0.0	2.5 ± 0.8	119.4 ± 3.9

4.7	2.3	0.0 ± 0.0	0.0 ± 0.0	112.3 ± 12.3
5.2	2.8	0.0 ± 0.0	0.0 ± 0.0	102.2 ± 10.4
5.7	3.3	0.0 ± 0.0	0.0 ± 0.0	93.8 ± 7.4
6.2	3.8	0.0 ± 0.0	2.2 ± 0.7	92.9 ± 6.2
6.7	4.3	0.0 ± 0.0	0.0 ± 0.0	89.2 ± 5.1
7	4.8	0.0 ± 0.0	0.0 ± 0.0	87.7 ± 5.1
8	5.3	0.0 ± 0.0	0.0 ± 0.0	86.8 ± 2.3
8	5.8	0.0 ± 0.0	0.0 ± 0.0	73.2 ± 3.2
12	9.6	43.1 ± 2.0	4.2 ± 0.7	46.4 ± 1.9
18	15.6	61.2 ± 2.4	24.9 ± 0.7	41.1 ± 3.8
24	21.6	74.1 ± 4.5	25.4 ± 2.8	30.5 ± 2.8
48	45.6	81.0 ± 5.7	20.8 ± 0.7	18.5 ± 1.1
72	69.6	80.2 ± 5.5	6.4 ± 0.4	17.1 ± 1.2
96	93.6	40.2 ± 1.9	3.2 ± 0.6	12.9 ± 0.7
120	117.6	17.9 ± 1.6	2.7 ± 0.7	12.5 ± 1.1
144	141.6	5.7 ± 0.9	3.9 ± 1.3	6.4 ± 0.5
168	165.6	4.1 ± 0.9	2.1 ± 0.4	2.0 ± 0.1
240	237.6	0.0 ± 0.0	1.6 ± 0.4	0.1 ± 0.0
264	261.6	0.0 ± 0.0	2.3 ± 0.5	0.1 ± 0.0
288	285.6	0.0 ± 0.0	1.9 ± 0.8	0.1 ± 0.0
312	309.6	0.0 ± 0.0	1.1 ± 0.2	0.3 ± 0.1
