# Supplementary material for: Pharmacokinetics of Snake Antivenom Following Intravenous and Intramuscular Administration in Envenomed Large Animal Model
Source: Pharmaceutics. 2025 Feb 7;17(2):212. doi: 10.3390/pharmaceutics17020212 (PMC11859798; doi:10.3390/pharmaceutics17020212)
Supplement: Supplementary file 1 [file pharmaceutics-17-00212-s001.zip › Supplementary Table S6.pdf]

Table S6. Pharmacokinetic parameteres of *s.c.* applied venom (m = 20 mg) and ammodytoxins (Atxs), as well as of *i.m.* ($S_{i.m.}$ group) and *i.v.* ($S_{i.v.}$ group) antivenoms (m = 400 mg) in the systemic circulation.

	**ANTIVENOM**					**VENOM**				**ATX**			
	**$S_{i.m.}$**		**$S_{i.v.}$**			**$S_{i.m.}$**		**$S_{i.v.}$**		**$S_{i.m.}$**		**$S_{i.v.}$**	
	**mean**	**median (IQR)**	**mean**	**median (IQR)**		**mean**	**median (IQR)**	**mean**	**median (IQR)**	**mean**	**median (IQR)**	**mean**	**median (IQR)**
$t_{1/2}$ [h]	41.9 ± 10.7	46.0 (19.9 – 59.8)	34.6 ± 2.7	33.2 (30.6 – 40.2)	$t_{1/2}$ [h]	90.6 ± 61.7	36.6 (16.3 – 218.9)	104.3 ± 69.6	44.0 (19.2 – 249.5)	112.2 ± 19.7	117.8 (72.3 – 146.5)	101.5 ± 28.1	100.5 (48.9 – 155.2)
t_{max} [h]	32.3 ± 7.9*	33.8 (17.3 – 45.8)	0.6 ± 0.2*	0.6 (0.3 – 1.0)	t_{max} [h]	1.7 ± 0.8	2.0 (0.9 – 2.2)	24.7 ± 13.5	24.9 (1.1 – 48.0)	1.5 ± 0.5	1.6 (0.6 – 2.3)	12.5 ± 6.7	12.6 (0.8 – 24.0)
c_{max} [µg mL^{-1}]	52.3 ± 11.6*	43.0 (37.4 – 76.6)	214.8 ± 36.6*	187.2 (166.6 – 290.7)	c_{max} [ng mL^{-1}]	61.7 ± 8.7	59.7 (46.6 – 78.9)	59.4 ± 10.7	62.2 (37.5 – 78.6)	11.0 ± 3.7	8.3 (5.9 – 18.9)	11.7 ± 4.9	9.3 (4.1 – 21.7)
V_z [L]	4.7 ± 1.8	3.8 (1.8 – 8.6)	4.2 ± 0.8	4.7 (2.6 – 5.2)	V_z [L]	744.0 ± 306.0	487.5 (365.1 – 1379.6)	371.3 ± 144.5	326.3 (119.7 – 668.1)	748.6 ± 345.6	474.2 (299.1 – 1472.5)	224.8 ± 93.5	190.2 (66.2 – 418.0)
MRT [h]	82.6 ± 8.2*	84.6 (66.1 – 97.2)	57.5 ± 2.6*	58.2 (52.5 – 62.0)	MRT [h]	149.1 ± 98.3	64.1 (32.4 – 350.9)	163.7 ± 91.2	82.6 (55.6 – 352.8)	176.5 ± 38.3	172.5 (106.4 – 250.5)	188.1 ± 57.3	168.8 (92.3 – 303.2)
AUC_{∞} [(mg mL^{-1}) · min]	358.0 ± 58.0	371.7 (240.3 – 462.1)	331.2 ± 86.8	266.6 (217.2 – 509.8)	AUC_{∞} [(µg · mL^{-1}) · min]	153.0 ± 49.6	130.3 (71.7 – 257.0)	399.4 ± 124.8	377.5 (169.9 – 650.8)	41.6 ± 11.3	45.7 (19.1 – 60.2)	68.4 ± 21.3	67.2 (30.1 – 107.9)
AUC_{0-t} [(mg mL^{-1}) · min]	354.8 ± 57.0	366.2 (240.2 – 458.0)	329.9 ± 86.5	265.3 (216.5 – 508.0)	AUC_{0-t} [(µg · mL^{-1}) · min]	95.2 ± 13.5	95.9 (68.8 – 120.9)	296.5 ± 72.0	300.9 (155.2 – 433.3)	23.5 ± 7.2	25.8 (8.6 – 36.0)	53.8 ± 20.5	45.2 (19.7 – 96.4)
CL [mL min^{-1}]	1.2 ± 0.2	1.1 (0.8 – 1.7)	1.4 ± 0.3	1.5 (0.9 – 1.9)	CL [mL min^{-1}]	178.4 ± 53.1	166.7 (82.3 – 286.2)	73.5 ± 27.7	56.7 (32.1 – 131.9)	87.9 ± 38.6	70.9 (25.4 – 167.5)	24.6 ± 8.1	23.4 (10.4 – 40.0)

*Significant difference between $S_{i.m.}$ and $S_{i.v.}$ group ($p < 0.05$).
